# A rubbery semiconducting heterojunction film for fully rubbery multiplexed near-infrared phototransistor arrays

**DOI:** 10.1126/sciadv.aee2602

**Published:** 2026-05-27

**Authors:** Yu-Dong Zhao, Na Li, Junmei Hu, Wei-Chen Gao, Ben Fan, Xiang Sun, Defeng Cui, Jing Qiao, Ying-Shi Guan

**Affiliations:** School of Chemistry and Chemical Engineering, Southeast University, Nanjing 211189, China.

## Abstract

High-sensitivity photodetecting semiconductors with intrinsic stretchability and excellent charge carrier collection efficiency are imperative for the advancement of rubbery phototransistors. However, the scalable manufacturing of such materials into high-performance, multiplexed rubbery near-infrared phototransistor arrays remains challenging. Herein, we report a highly doped, rubbery semiconducting nanofilm with a unique nanoweb structure, fabricated by precisely controlling the composition of a ternary blend, comprising a semiconducting polymer (DPP-TT), a molecular dopant (F4TCNQ), and an elastic matrix (polyurethane). This unique architecture provides both high charge carrier mobility and exceptional mechanical stretchability. The developed rubbery phototransistors and their multiplexed arrays maintain electrical functionality even under 50% strain. Notably, the multiplexed phototransistor array maintains high imaging fidelity, capable of reproducing well-defined patterns even in deformed states. Furthermore, we demonstrate the application of this imaging system as a two-dimensional stretchable barcode, leveraging its multichannel, illumination-dependent responses. This work provides a viable pathway for the scalable production of high-performance rubbery optoelectronics.

## Teaser

A customized rubbery semiconducting heterojunction film with desired photoresponse enables stretchable optoelectronic barcodes.

## INTRODUCTION

Cutting-edge near-infrared (NIR) photodetector imaging systems hold considerable promise in the field of intelligent visualization, with applications spanning from night-vision equipment and nondestructive inspection to proactive health monitoring and optical communication networks (*1*–*5*). The advent of the Fourth Industrial Revolution is driving a critical need for seamless interconnection and communication between humans and machines. In this context, highly sensitive and rubbery NIR photodetectors are essential for bridging human-machine interfaces, thereby facilitating the development of next-generation interactive systems. Driven by the burgeoning demand for soft electronics, rubbery photodetector arrays that can accommodate repetitive mechanical deformations while accurately sensing high-quality signals have attracted intense research interest (*6*–*9*). However, existing deformable NIR photodetectors face critical challenges. Conventional high-performance devices rely on rigid inorganic semiconductors (e.g., silicon and InGaAs), which achieve excellent detectivity and fast response time but are fundamentally brittle and incompatible with stretchable form factors (*10*). To impart mechanical compliance, researchers have used strain-dissipative structural designs, such as nonplanar pop-up geometries, serpentine interconnects, and kirigami-based structures, with these inorganic materials (*11*–*13*). While effective in preventing fracture, these approaches introduce cumbersome fabrication processes and limit pixel density due to the redundant interpixel spacing required to accommodate deformation, ultimately constraining device resolution and scalability (*14*).

Semiconducting polymers offer a compelling alternative due to their intrinsic flexibility, tailorable molecular structures, and cost-effective solution processability (*15*–*17*). A central challenge, however, lies in the inherent trade-off between electrical functionality and mechanical ductility: highly ordered crystalline domains facilitate efficient charge transport, while amorphous regions are essential for stretchability (*18*–*21*). Incorporation of trace amounts of molecular dopants has emerged as an appealing route to concurrently augment stretchability by expanding the amorphous regions and enhance electrical conductivity by diminishing the hopping barrier and suppressing trap states (*22*, *23*). However, critical hurdles still remain, namely, the insufficient operation resilience and durability under high-strain repetitive stress. Furthermore, integrating conjugated polymers with soft elastomer matrix for rubber-like polymer composites has demonstrated a highly viable pathway (*24*–*28*). The synergy of physical nanoconfinement and chemical doping holds considerable potentials for advancing next-generation optoelectronics, benefitting from the inherent compatibility of molecular dopants with polymeric systems. Although conceptually simple, the growth of large-scale and uniform rubbery semiconducting nanofilms remains a challenge, primarily due to the difficulty in precisely controlling their chemical composition and optoelectrical properties, from the bottom up.

Here, we report a highly doped rubbery semiconducting nanofilm and a multiplexed rubbery NIR phototransistor array fabricated via the air/water interfacial self-assembly process. This demonstrated that a reliable production technique enables the large-area and scalable fabrication of a ternary system comprising the semiconducting polymer poly{2,5-(2-octyldodecyl)-3,6-diketopyrrolopyrrole-alt-5,5-[2,5-di(thien-2-yl)thieno [3,2-b]thiophene]} (DPP-TT), molecular dopant 2,3,5,6-tetrafluoro-7,7,8,8-tetra-cyanoquinodimethane (F4TCNQ), and elastic polymer matrix polyurethane (PU). Chemical redox reactions triggered by F4TCNQ within concentration-controlled solutions enable the precise definition of doping level to achieve high photosensitivity. The resulting rubbery semiconducting nanofilm has a unique nanoweb structure, featuring finely tuned edge-on stacking with minimal chain disruption. This structure provides both geometrical stretchability and high interconnectivity required for efficient charge injection and transport. Rubbery phototransistors and their multiplexed arrays have been successfully fabricated using the rubbery semiconducting nanofilm. Rubbery phototransistors can retain their electrical functionality when stretched to 50% strain and the multiplexed phototransistor arrays can accurately detect NIR patterns for multichannel mapping in deformed states. Furthermore, leveraging their multichannel coupling and illumination-dependent responses, the imaging system demonstrates potential as a two-dimensional (2D) stretchable barcode for advanced signal processing. Our work has provided a deeper comprehension of constructing high-performance rubbery semiconducting nanofilm and contributed to realizing multifunctional optoelectronics.

## RESULTS

### Rubbery semiconducting nanofilm from a ternary system

**Fig. 1.**
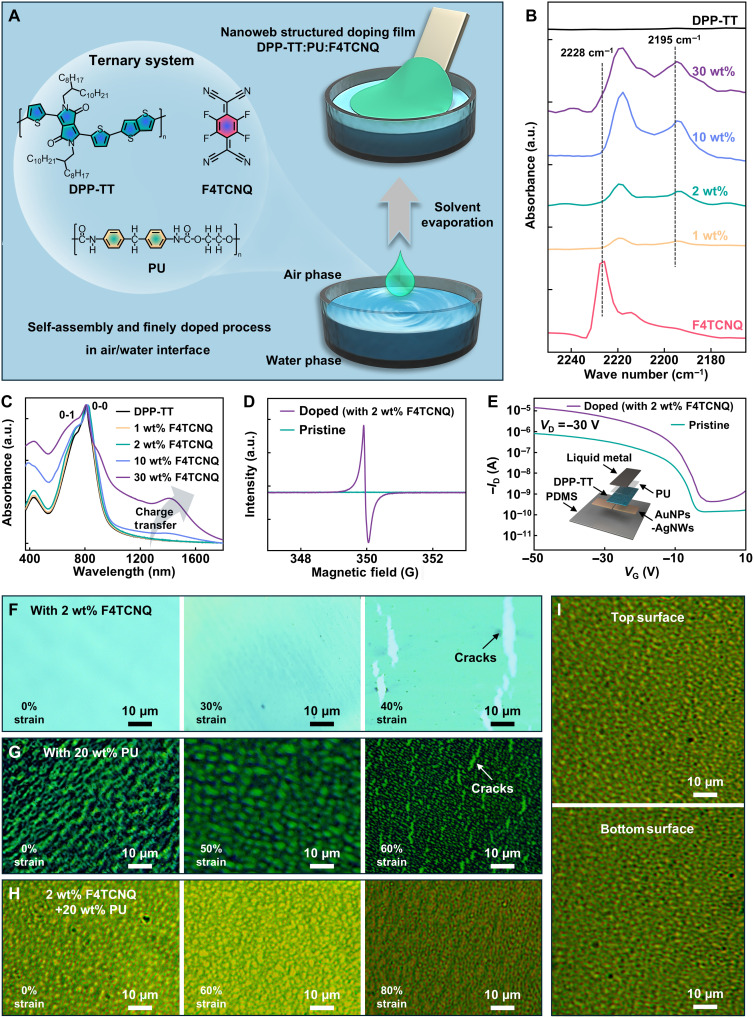
The air/water interface–assembled DFP nanofilm. (**A**) Chemical structures of DPP-TT, F4TCNQ, and PU, as well as schematic representation of the DFP nanofilm through the air/water interfacial self-assembly. (**B** to **D**) Description of charge transfer effect. (B) FTIR spectra of pristine DPP-TT films, pristine F4TCNQ films, and doped DPP-TT films with different F4TCNQ concentrations. (C) UV-vis-NIR absorption spectra of pristine and doped DPP-TT polymer films with different F4TCNQ concentrations. (D) EPR analysis of pristine and doped DPP-TT (2 wt % F4TCNQ) polymer solutions. (**E**) Representative transfer curves of the thin-film transistors based on pristine and doped DPP-TT (2 wt % F4TCNQ). The inset shows the transistor device architecture. (**F**) The optical images of the blended DPP-TT nanofilms with 2 wt % F4TCNQ stretched at 0, 30, and 40% strain, respectively. (**G**) The optical images of the blended DPP-TT nanofilms with 20 wt % PU stretched at 0, 50, and 60% strain, respectively. (**H**) The optical images of the blended DPP-TT nanofilms with 2 wt % F4TCNQ and 20 wt % PU stretched at 0, 60, and 80% strain, respectively. (**I**) The optical images of the top and bottom surfaces of the DFP nanofilm.

The air/water interfacial self-assembly of a rubbery semiconducting nanofilm with efficient p-doping is schematically depicted in Fig. 1A. This process is achieved by combining a widely accessible dopant, a semiconducting polymer, and an elastomer. Specifically, the readily available p-dopant F4TCNQ, known for its compatibility with semiconductor devices, was used to modulate charge carrier transport. The semiconducting polymer DPP-TT was selected for its ability to form a percolated network with F4TCNQ, enabling efficient charge transport. Last, PU was applied as an elastomer matrix to impart stretchability. Driven by the Marangoni effect (*29*, *30*), a drop of the blended solution propagates immediately across the water surface, causing the rapid vaporization of the solvent and the formation of a large-area freestanding rubbery semiconducting nanofilm (fig. S1). This facile manufacturing strategy produces a uniform, high-yield rubbery semiconducting nanofilm that is readily transferable onto diverse elastic or rigid substrates (fig. S2). The simple manufacturing process yields a 25 × 10 cm^2^ area without relying on complex instrumentation (fig. S3). We have further demonstrated the general applicability of an air/water interfacial self-assembly method on other polymeric semiconductors, which exhibits ideal transfer curves (fig. S4).

The chemical doping methodology via the incorporation of a dopant molecule into the host semiconducting material is straightforward and efficient, as demonstrated in Fig. 1 (B to D) for the case study of charge transfer between the DPP-TT and F4TCNQ. Notably, the representative aromatic molecule of DPP-TT with extensive π-conjugated structure exhibits negative electrostatic potential (ESP) values, indicating its strong electron repulsion nature. Conversely, the typical p-type dopant F4TCNQ demonstrates positive ESP values, confirming its powerful electron-withdrawing property (fig. S5). Furthermore, the neutral DPP-TT with an ionization energy (IE^0^) ~5.2 eV can donate an electron to neutral F4TCNQ, which has an electron affinity (EA^0^) ~5.2 eV (fig. S6). This similar offset is favorable for formation of active radicals with the unpaired electrons through complete charge transfer. In this regard, DPP-TT and F4TCNQ were rationally used as the corresponding host and dopant (*31*, *32*). The characteristic stretching frequencies of the cyano groups (C≡N) in F4TCNQ were identified by Fourier transform infrared (FTIR) spectra to gain insight into the redox states of dopant molecule and the relative numbers of anions by charge transfer (Fig. 1B) (*32*). Noticeably, the C≡N stretching vibration shifts from 2228 cm^−1^ for neutral F4TCNQ to 2195 cm^−1^ for the F4TCNQ anion, with the anion peak exhibiting different intensities at varying concentrations in the doped polymer films. The doping level increases with the concentration of F4TCNQ, which was varied from 1 to 30 wt %. We further conducted the ultraviolet–visible–near-infrared (UV-vis-NIR) absorption spectra to evaluate the generated charge carriers in DPP-TT films upon doping (Fig. 1C). An intense polaronic absorption extending in the “sub-bandgap” region (1000 nm) is observed compared with the pristine DPP-TT film, especially in higher concertation of F4TCNQ doping condition, which derived from the ionized DPP-TT chains and F4TCNQ anions (*33*). Similarly, the emerging peak at 1420 nm attributed to charge transfer interaction evolves positively with increasing doping concentration of F4TCNQ (*34*). Noticeably, the characteristic peaks at 730 and 810 nm of pristine DPP-TT correspond to 0-1 and 0-0 vibronic transitions, respectively (*33*). The low-energy 0-0 transition peak associated with short-range order aggregation is relative weakened as the doping concentration of F4TCNQ increases, contributing to the enhanced stretchability of semiconducting polymer (*35*). The electron paramagnetic resonance (EPR) characterization was also performed to test the polarons generated in the doped polymers (Fig. 1D). Even at low F4TCNQ concentrations (1 and 2 wt %), notable EPR signals were observed, in contrast to the negligible signals from the pristine DPP-TT and F4TCNQ materials (fig. S7). The simultaneous presence of both DPP-TT radical cations and F4TCNQ radical anions at low doping concentrations illustrates the efficient doping process. Furthermore, the field-effect mobility of the highly tunable doping DPP-TT films was investigated based on thin-film transistor devices. The device features a high-mobility channel layer (semiconducting polymer DPP-TT doped with different concentrations of F4TCNQ), low-contact-resistance source-drain electrodes [gold nanoparticle (AuNP)–coated silver nanowires (AgNWs), AuNPs-AgNWs], an elastic dielectric material (PU), and a smooth gate electrode [eutectic liquid metal alloy of gallium-indium (liquid metal)] (inset of Fig. 1E). The used AuNPs-AgNWs/polydimethylsiloxane (PDMS) composites create a relatively decreased resistance contact with DPP-TT attributed to the higher work function of AuNPs (fig. S8). The representative transfer and output characteristics of the transistors based on pristine DPP-TT and doped DPP-TT (2 wt % F4TCNQ) are displayed in Fig. 1E and fig. S9. The notable enhancement in mobility from 0.015 to 1.00 cm^2^ V^−1^ s^−1^ and the impressive increase in on/off ratio from 4.5 × 10^3^ to 3.4 × 10^4^ are attributed to the F4TCNQ, which considerably passivates charge traps through p-type doping (*36*). Substantially, the average mobility obtained from different doping concentrations is 0.0561 cm^2^ V^−1^ s^−1^ (0 wt % F4TCNQ), 0.589 cm^2^ V^−1^ s^−1^ (1 wt % F4TCNQ), 0.904 cm^2^ V^−1^ s^−1^ (2 wt % F4TCNQ), 0.510 cm^2^ V^−1^ s^−1^ (5 wt % F4TCNQ), 0.399 cm^2^ V^−1^ s^−1^ (10 wt % F4TCNQ), and 0.317 cm^2^ V^−1^ s^−1^ (30 wt % F4TCNQ). The on/off ratio increases with F4TCNQ concentration up to 2 wt %; however, higher concentrations induce phase separation and structural disorder, degrading charge transport owing to excessive doping (figs. S10 and S11). Therefore, the optimum electronic performance is found to be strongly correlated with the F4TCNQ concentration of 2 wt %. Despite increased surface roughness and structural disorder from excessive dopants, the preserved continuity of the semiconductor fibrous network still promises charge transport for 30 wt % F4TCNQ doping, attributed to the dopant stability/processability and counterion-semiconductor miscibility (fig. S12, A to C). However, more dopants likely induce destruction of current percolation pathways, leading to the observed permanently “on state” at 40 and 50 wt % doping (fig. S12D). Furthermore, ultraviolet photoelectron spectroscopy (UPS) analysis showed that the Fermi level (*E*_F_) of DPP-TT doped with 2 wt % F4TCNQ experienced a large shift by 1.02 eV toward the highest occupied molecular orbital (HOMO; fig. S13). Notably, F4TCNQ-induced p-doping lowers the hole injection barrier by shifting the *E*_F_ closer to the HOMO level, while the replacement of AgNWs with AuNPs minimizes the contact resistance at the metal-semiconductor interface, collectively enabling effective charge injection.

Furthermore, the stretchability of the DPP-TT–based blended semiconductor nanofilm, fabricated by the air/water interfacial self-assembly, was specially investigated and optimized. Actually, the addition of F4TCNQ could endow semiconducting polymer a certain compliance ascribed to the impact on ordering of DPP-TT, as supported by the UV-vis-NIR results (Fig. 1C). The fracture initiation strain of the blended films was tuned by systematically modulating the weight ratio of F4TCNQ to DPP-TT (fig. S14). Prominently, the maximum mechanical stretchability coincides with the optimal electronic behavior at 2 wt % F4TCNQ. Nevertheless, this blended film could maintain its ductility and compliance only up to 40% strain (Fig. 1F). Introducing a certain amount of PU to DPP-TT can improve its stretchability, as it inherits the elastic mechanical properties of the PU matrix. Rubbery semiconducting nanofilms with a nanoweb structure, formed at PU percentages of 20, 40, and 60 wt %, exhibit both geometrical stretchability and the high interconnectivity required for current flow pathways (fig. S15). This structure provides excellent elasticity and high fracture energy, facilitating efficient energy dissipation under strain (*37*). Specifically, we achieved larger pore diameters by increasing the PU content, resulting in enhanced stress tolerance and crack onset strain (fig. S16). Leveraging the excellent stress-dissipating elasticity of the PU matrix, the unique microphase-separated nanoweb structure could help to release applied strain and preserve crystalline domains, thereby safeguarding charge transport from damage. The rubbery semiconducting nanofilm with 20 wt % PU exhibits superior charge transport characteristics in the series (figs. S17 and S18), while visible cracks appear when it is stretched to 60% strain (Fig. 1G). By combining the advantages of PU and F4TCNQ, we achieved a highly reliable realization of mechanical stretchability alongside a notable improvement in electronic performance (Fig. 1H). The resulting rubbery semiconducting nanofilm comprising 2 wt % F4TCNQ and 20 wt % PU (denoted as DFP nanofilm) was fabricated through air/water interfacial assembly demonstrating strain-tolerance capacity without obvious crack formation even when stretched to 80% strain. The identical nanoweb characteristic is clearly detected in both the top and bottom surface topographic structures of the DFP nanofilm, implying no vertical phase separation (Fig. 1I). Critically, the doping level and mechanical stretchability could be regulated by simply changing the ratio of F4TCNQ and PU in the DPP-TT solution, affording distinguished scalability and tunability.

**Fig. 2.**
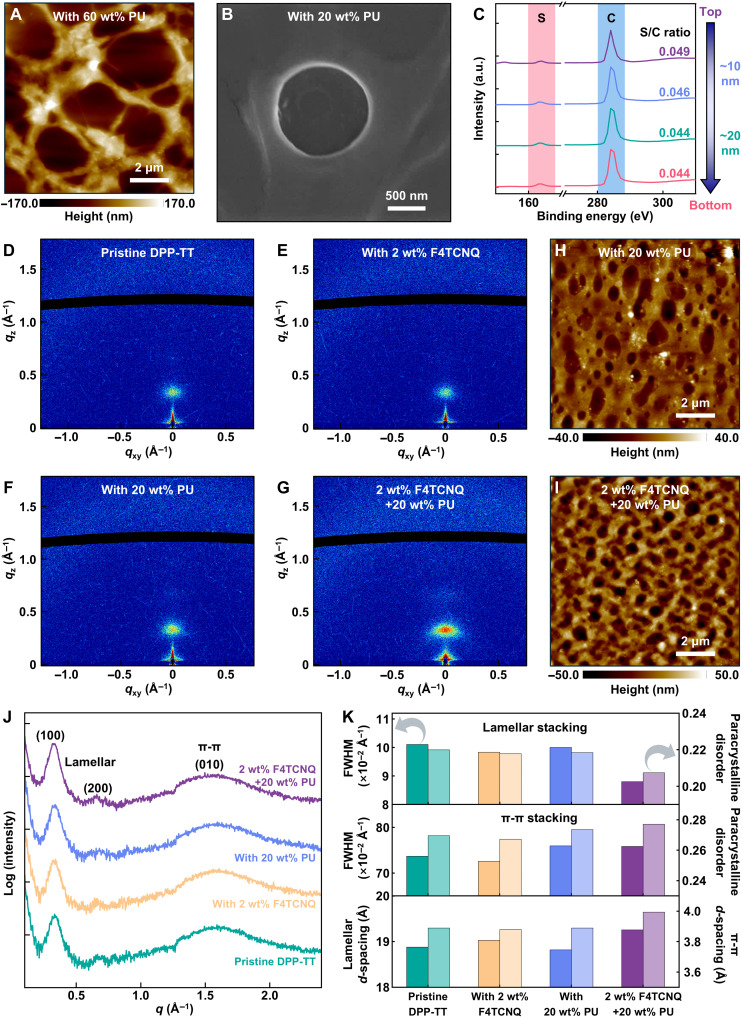
Characterization of DFP nanofilm. (**A**) AFM height image of the rubbery semiconducting nanofilm with 60 wt % PU. (**B**) The SEM image of the rubbery semiconducting nanofilm (20 wt % PU) acquired after sputter-coating with a gold layer to enhance its conductivity. (**C**) XPS spectra at different depths of the rubbery semiconducting nanofilm with 20 wt % PU. (**D** to **G**) 2D GIWAXS patterns of the spin-coated pristine DPP-TT nanofilm (D), the spin-coated DPP-TT nanofilm doped with 2 wt % F4TCNQ (E), the rubbery DPP-TT nanofilm with 20 wt % PU (F), and the DFP nanofilm with 20 wt % PU and 2 wt % F4TCNQ (G). (**H** and **I**) AFM height images of the rubbery DPP-TT nanofilm with 20 wt % PU (H) and the DFP nanofilm with 20 wt % PU and 2 wt % F4TCNQ (I). (**J**) The corresponding 1D GIWAXS profiles of the spin-coated pristine DPP-TT nanofilm, the spin-coated DPP-TT nanofilm doped with 2 wt % F4TCNQ, the rubbery DPP-TT nanofilm with 20 wt % PU, and the DFP nanofilm with 20 wt % PU and 2 wt % F4TCNQ. (**K**) The summarized FWHM, paracrystalline disorder, and packing distance of lamellar stacking and π-π stacking. In particular, all the rubbery nanofilms with PU were fabricated through the air/water interfacial self-assembly method. In contrast, nanofilms without PU were constructed by spin coating.

To elucidate the spatially morphological features of the rubbery semiconducting nanofilm composed of PU and DPP-TT, atomic force microscopy (AFM) and scanning electron microscopy (SEM) images, together with in-depth elemental analysis by x-ray photoelectron spectroscopy (XPS) were performed. Notably, the DPP-TT semiconducting polymers phase-separate into nanoconfined fibers ascribed to strong π-π interactions between DPP-TT molecules (*24*–*26*). The unique nanoconfinement effect could suppress conformational disorder, thereby yielding satisfactory carrier mobility despite the introduction of insulating elastomer PU (*37*). The resulting microphase-separated nanoweb structure observed in rubbery semiconducting nanofilm is driven by a gradient in surface energy between DPP-TT and PU. AFM images of rubbery semiconducting nanofilms with varying PU fractions show that the nanoweb structure becomes progressively slenderer, with larger holes, as the PU content increases from 20 to 60 wt % (Fig. 2A and fig. S19). This arises from the inhibited crystal nucleation and growth of the semiconducting polymer, constrained within the PU elastic matrix (*15*). Furthermore, by precisely controlling the PU content and the solution volume per addition, a microphase-separated nanoweb structure can be reliably reproduced at room temperature in our system (figs. S20 and S21). Corresponding SEM images corroborate the AFM findings, revealing the same nanoweb structure (Fig. 2B and fig. S22). The nanoweb structure was further probed along the nanofilm’s thickness direction using XPS depth profiling (Fig. 2C). XPS spectra were acquired at different depths (top, ~10 nm, ~20 nm, and bottom) of the rubbery semiconducting nanofilm (20 wt % PU). Notably, intense and similar sulfur (S) XPS core level signals from the DPP-TT semiconducting polymer were observed at all four profiling points. The nearly identical S 2*p* to carbon (C)1*s* intensity ratio across these depths strongly confirms that DPP-TT is uniformly distributed throughout the entire nanofilm.

The impact of F4TCNQ dopants on the crystalline packing behavior of DPP-TT was revealed using grazing-incidence wide-angle x-ray scattering (GIWAXS) measurements. The similar 2D diffraction patterns observed for all films demonstrate a semicrystalline lamellar structure with an edge-on molecular orientation, suggesting that the addition of F4CTNQ (2 wt %) does not disrupt the predominant chain stacking of DPP-TT (Fig. 2, D to G, and fig. S23). Furthermore, the analogous AFM images of the rubbery semiconducting nanofilm (20 wt % PU) and its doped counterpart (20 wt % PU, 2 wt % F4TCNQ), combined with their nearly identical 2D GIWAXS patterns, attest to a nondestructive doping mechanism (Fig. 2, H and I). The corresponding GIWAXS 1D profiles are shown in Fig. 2J. Obvious (*h*00) diffraction peaks originating from the lamellar stacking are recognized in the low-*q* region, and the (010) diffraction peak arising from the π-π packing is identified in the high-*q* region. The lamellar (*h*00) packing distance of DPP-TT films gradually increases with F4TCNQ doping concentration, from 18.88 Å (pristine) to 19.03 Å (2 wt % F4TCNQ) and 19.74 Å (10 wt % F4TCNQ) (Fig. 2K and figs. S24 and S25). The π-π (010) packing spacing decreases from 3.89 Å (pristine) to 3.84 Å (2 wt % F4TCNQ), but increases to 3.90 Å (10 wt % F4TCNQ). The observed decrease in π-π distance coupled with an expanded lamellar spacing implies that a small amount of F4TCNQ (2 wt %) is predominantly accommodated in the side chains, thereby fine-tuning the microstructure without disrupting the overall chain alignment. The absence of broadening in the corresponding full width at half maximum (FWHM), along with the decrease in the disorder degree of both the lamellar and π-π stacking after doping, further confirms the nondestructive nature of the doping process (Fig. 2K). The proposed nondestructive doping concept involves the intercalation of F4TCNQ into the polymer lamellar structures without disrupting the crystalline order, leading to preserved and even improved overall crystallinity (fig. S26). However, the excessive dopant molecule F4TCNQ (10 wt %) could locate in the π-π stacking region, leading to the increment of π-π spacing and destruction on the polymer packing (*38*). The optimal doping concentration of 2 wt % is attributed to its preservation of a nondestructive solid-state microstructure during the moderate doping process, thereby yielding the best electronic performance (figs. S11 and S27). Regarding the rubbery semiconducting nanofilm (20 wt % PU) and its doped counterpart (20 wt % PU and 2 wt % F4TCNQ), the improved lamellar distance from 18.82 to 19.26 Å strongly indicates the successful insertion of F4TCNQ, together with the diminished FWHM and paracrystalline disorder. Conversely, the increased π-π spacing from 3.89 to 3.99 Å as well as the enlarged FWHM and paracrystalline disorder could account for the enhanced stretchability by increasing structural disorder (*33*). Consequently, the combination of physical nanoconfinement and chemical redox reactions during air/water interfacial self-assembly yields highly doped omnidirectional percolation nanoweb architecture, which simultaneously have notably improved stretchability and substantially enhanced electronic performance.

### Rubbery transistor arrays

**Fig. 3.**
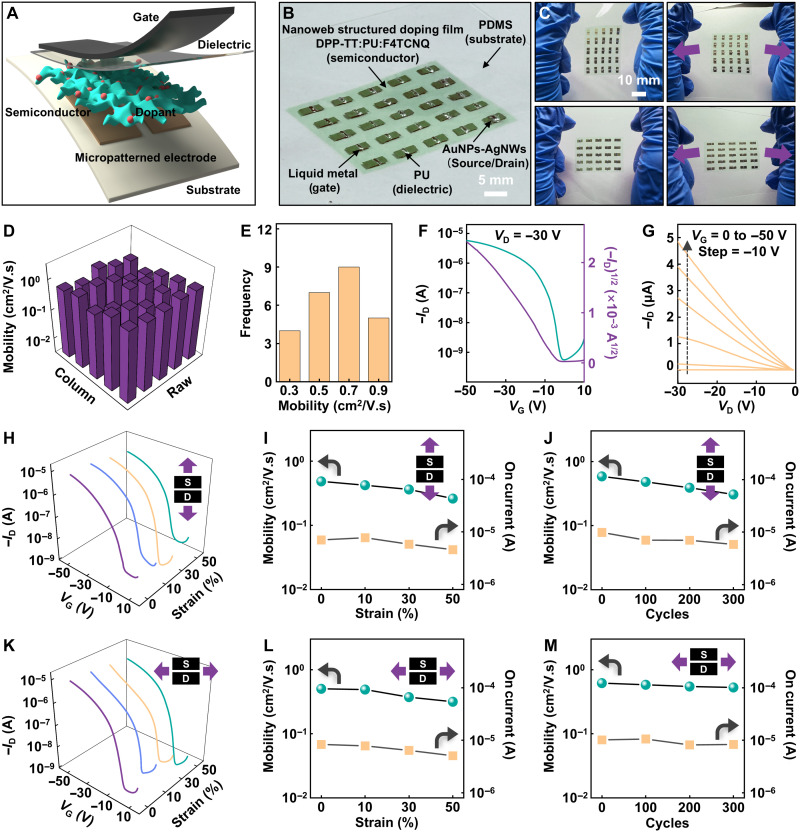
Electronic performance and mechanical stretchability of the rubbery transistor array. (**A**) Diagram showing the rubbery transistor. (**B**) The optical image of the fabricated fully rubbery transistor array. (**C**) The optical images of deformed fully rubbery transistor array under tensile strain modes both along and perpendicular to the channel length direction. (**D**) Calculated field-effect mobility map of the rubbery transistor array. (**E**) Histogram showing mobilities from the 25 working transistors in the 5 × 5 rubbery transistor array. (**F** and **G**) Representative transfer curve (F) and output curve (G) of the rubbery transistors. (**H**) Typical transfer curves of the rubbery transistor stretched at mechanical strains of 0, 10, 30, and 50% along the channel length. (**I**) Changes in mobility during stretching to 50% strain along the channel length. (**J**) Changes in mobility after stretch-release cycles at 30% strain along the channel length. (**K**) Typical transfer curves of the rubbery transistor stretched at mechanical strains of 0, 10, 30, and 50% in perpendicular direction to the channel length. (**L**) Changes in mobility during stretching to 50% strain in perpendicular direction to the channel length. (**M**) Changes in mobility after stretch-release cycles at 30% strain in perpendicular direction to the channel length.

Harnessing the advantages of the highly doped rubbery DFP nanofilm, we developed fully rubbery transistors following the device configuration depicted in Fig. 3A. A soft, elastic 5 × 5 rubbery transistor array was successfully fabricated (Fig. 3B). Optical images in Fig. 3C show the array under tensile strain, applied both parallel (top) and perpendicular (bottom) to the channel length. The array withstands the pressure from the forceps reflecting the excellent mechanical resilience (fig. S28). All the rubbery transistors based on the DFP nanofilm perform well with a yield of 100%, which demonstrate reliable transfer characteristics (fig. S29A). As further revealed by AFM and XRD (figs. S30 and S31), the microphase-separated nanoweb structure and molecular packing of the DFP nanofilms both exhibit high spatial uniformity over the entire fabricated area. The high uniformity, essential for integrated electronics, is demonstrated by the field-effect mobility map and its statistical distribution (Fig. 3, D and E). The representative transfer and output curves of the rubbery transistor are displayed in Fig. 3 (F and G). The highest and average carrier mobilities are 0.938 and 0.625 cm^2^ V^−1^ s^−1^, respectively. Moreover, the device shows fairly uniform performance with an average on/off ratio approximately of 10^4^ (fig. S18B). The electronic performance is well maintained without substantial damage when stretched either along or perpendicular to the channel length direction. The fabricated rubbery transistor exhibits excellent compliance and resilience, maintaining functionality even when subjected to uniaxial strain as high as 50%, as shown by the transfer curves in Fig. 3 (H and K). When stretched along the channel length direction (Fig. 3I), the mobility decreases slightly from 0.483 cm^2^ V^−1^ s^−1^ (0% strain) to 0.421 cm^2^ V^−1^ s^−1^ (10% strain), 0.362 cm^2^ V^−1^ s^−1^ (30% strain), and 0.261 cm^2^ V^−1^ s^−1^ (50% strain). When stretched perpendicular to the channel length directions (Fig. 3L), the mobility shows only a minor degradation, changing from 0.503 cm^2^ V^−1^ s^−1^ (0% strain) to 0.489 cm^2^ V^−1^ s^−1^ (10% strain), 0.373 cm^2^ V^−1^ s^−1^ (30% strain), and 0.316 cm^2^ V^−1^ s^−1^ (50% strain). The minimal variation in drain current, regardless of stretching direction, confirms its strain-insensitive electrical behavior. Furthermore, both the drain current and carrier mobility show no obvious degradation during cyclic stretching tests in both directions (up to 300 cycles at 30% strain), demonstrating the exceptional durability of the rubbery transistor array (Fig. 3, J and M, and fig. S32). To fully demonstrate the robustness under cyclic stretching, the fabricated rubbery transistors were subjected to 2000 stretching-releasing cycles at 30% strain along or perpendicular to the channel length direction (fig. S33). Notably, our proposed strategy specifically aims to bridge the critical gap in achieving a careful balance among device uniformity, large-area fabrication, stretchability, and mobility for high-performance stretchable electronics (table S1). The enhancement in carrier mobility is predominantly driven by F4TCNQ redox doping, while the superior mechanical stretchability is primarily due to the PU nanoconfinement effect. F4TCNQ doping exerts a more pronounced influence on modulating the threshold voltage (table S2 and fig. S34).

### High-sensitivity NIR response of rubbery phototransistors

**Fig. 4.**
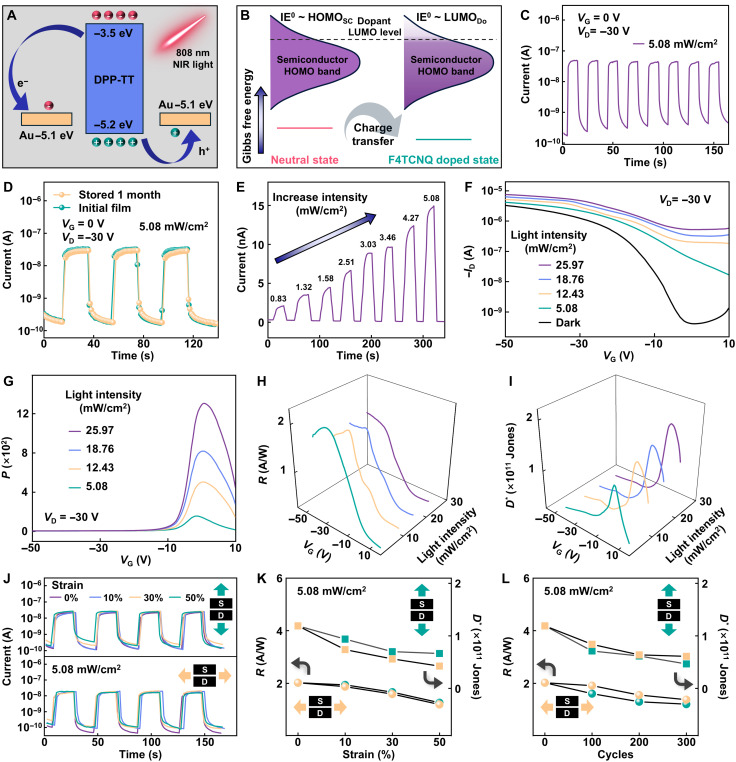
Rubbery NIR detector based on the DFP nanofilm. (**A**) Schematic showing carriers movement and the corresponding energy band diagrams under 808-nm illumination. (**B**) The energy alignment diagram depicting the p-type chemical doping process between the semiconductor (host) and the dopant molecule. The IE^0^ of the neutral organic semiconductor is equal to the edge of its semiconductor HOMO band (HOMO_SC_). LUMO_Do_, the LUMO level of the dopant. (**C**) NIR switching characteristics of the rubbery DFP nanofilm under the illumination of 5.08 mW/cm^2^ at *V*_D_ = −30 V (*V*_G_ = 0 V). (**D**) NIR temporal response of the rubbery DFP nanofilm stored for 1 month under the illumination of 5.08 mW/cm^2^ at *V*_D_ = −30 V (*V*_G_ = 0 V). (**E**) NIR temporal response of the rubbery DFP nanofilm under different illumination intensities from 0.83 to 5.08 mW/cm^2^ at *V*_D_ = −30 V (*V*_G_ = 0 V). (**F**) Typical transfer characteristics of the rubbery DFP phototransistor conducted in the dark and under the illumination of 5.08 mW/cm^2^ at *V*_D_ = −30 V. (**G** to **I**) The corresponding *P* (G), *R* (H), and D^*^ (I) of the rubbery DFP phototransistor under various incident illumination intensities at *V*_D_ = −30 V. (**J**) NIR switching characteristics of the rubbery DFP nanofilm under the illumination of 5.08 mW/cm^2^ stretched at mechanical strains of 0, 10, 30, and 50% along or perpendicular to the channel length directions. (**K**) Changes in *R* and D^*^ under the illumination of 5.08 mW/cm^2^ during stretching to 50% strain along or perpendicular to the channel length directions. (**L**) Changes in *R* and D^*^ under the illumination of 5.08 mW/cm^2^ after stretch-release cycles at 30% strain along or perpendicular to the channel length directions.

As a proof of concept for fully deformable digital imaging, we comprehensively evaluated the NIR response functionality and stability of the fabricated rubbery transistors. Figure 4A illustrates that the mutually matched energy levels between the Au electrodes and DPP-TT polymers are beneficial for the exciton dissociation and carrier collection in the semiconducting channel (*15*). Noticeably, the energy alignment and change in Gibbs free energy indicate the favorable electrochemical redox potential between the DPP-TT host and the F4TCNQ dopant (Fig. 4B). The dopant accepts an integer number of electrons from the host via charge-transfer interactions in the ground state, which is known as the Marcus theory (*39*). The electrons within the HOMO band of the DPP-TT polymer can readily transfer to the lowest unoccupied molecular orbital (LUMO) band (dotted line) of the F4TCNQ molecule, so that the IE^0^ could align closely with the LUMO level of the dopant (Fig. 4B and fig. S35) (*40*). In the DFP nanofilm, the removal of electrons from the HOMO enables efficient hole injection and extraction, a notable improvement over the pristine DPP-TT polymer. The resulting increased hole concentration in the semiconducting channel lowers the effective barrier height, thereby enhancing charge tunneling and transport from the active layer to the Au electrodes to yield a higher photocurrent (*41*). The energy-level diagram of the heterojunction between DPP-TT and F4TCNQ depicted in fig. S36 illustrates the corresponding photogenerated carrier transfer processes under NIR illumination. Noticeably, the achieved heterojunction substantially facilitates the efficient processes of exciton generation, dissociation, and transport.

The photoresponse of the rubbery phototransistor under repeated NIR switching pulses under the illumination of 5.08 mW/cm^2^ is shown in Fig. 4C, which was conducted at *V*_G_ = 0 V to minimize the dark current and mitigate the influence of mobile charges. Upon illumination of the semiconductor layer, the rapid photon absorption generates a transient photocurrent that subsequently decays to an equilibrium state under electric field redistribution, yielding a high current on/off ratio of ~10^2^. The enhanced current on/off ratio (compared to ~27 for the pristine polymer) is primarily attributed to the increased carrier mobility, reduced interface defects, and improved contact between the nanoconfined semiconductor layer and the source/drain electrodes (fig. S37) (*15*). The rubbery phototransistor exhibits prominent electrical stability, characterized by no degradation in NIR detection properties after 1 month of storage (Fig. 4D). The retention of both the interlayer spacing from GIWAXS patterns (fig. S38) and the microscopic morphology from AFM characterization (fig. S39) after long-term storage sufficiently demonstrates the excellent doping stability. This long-term stability of the highly doped rubbery semiconducting nanofilm results from the effective liquid-phase charge transfer, which ensures compatibility with the polymer semiconductors by avoiding dopant aggregation during polymer self-assembly and preventing subsequent diffusion. As shown in Fig. 4E, the photocurrent responds sensitively to low illumination intensities, increasing linearly with illumination intensity from 0.83 to 5.08 mW/cm^2^ due to the rising concentration of photogenerated charge carriers (fig. S40). The transfer characteristics of the rubbery phototransistors under different illumination intensities show an ascending drain current and a positive shift in threshold voltage (*V*_th_), highlighting the pivotal role of the photogating effect in controlling photocarrier trapping within the semiconducting channel (Fig. 4F). The observed shift is ascribed to the photovoltaic effect, stemming from the combined action of photogenerated holes increasing the channel hole concentration and photogenerated electrons compensating for positively charged trap states (*16*). The rubbery phototransistor exhibits typical p-type output characteristics both in the dark and under illumination at 5.08 mW/cm^2^ (fig. S41). We evaluated the optoelectronic performance of the rubbery phototransistor using standard figures of merit including the photosensitivity (*P*), photoresponsivity (*R*), detectivity (D^*^) and external quantum efficiency (EQE) (Supplementary Text). All the corresponding parameters of fabricated rubbery NIR detectors are tunable by the gate bias and are sensitive to light intensity (Fig. 4, G to I, and fig. S42). Under the illumination of 25.97 mW/cm^2^, the rubbery NIR detector reaches a high *P* value up to ~10^3^. The *R* and EQE follow an opposite trend, decreasing with the increased illumination intensities ascribed to the carrier recombination and photon-electron converse saturation. The extracted maximum *R* and EQE are 2 A/W and 310% under the illumination of 5.08 mW/cm^2^, respectively. Furthermore, D^*^ exceeds 10^11^ Jones regardless of NIR illumination intensities. The NIR detection properties of the rubbery phototransistor under mechanical deformations were further verified (Fig. 4, J to L). The rubbery phototransistor exhibits excellent mechanical robustness, with its periodic on/off NIR response showing no notable degradation under applied strains of up to 50%, both parallel and perpendicular to the channel length (Fig. 4J). The summarized photoresponse times (*t*_Rise_ and *t*_Delay_) also demonstrate minimal changes when subjected to different strains (fig. S43). The uniaxial stretching tests on the rubbery phototransistor under 5.08 mW/cm^2^ illumination show that the *R* and D^*^ exhibit acceptable attenuation under strains of up to 50%, irrespective of the stretching directions (Fig. 4K). Cyclic stretching tests (up to 300 stretching and releasing cycles at 30% strain in both directions) demonstrate that the rubbery phototransistor exhibits excellent elastic recovery, showing no permanent plastic deformation (Fig. 4L). To comprehensively illustrate the robustness under cyclic stretching, the fabricated stretchable NIR phototransistors were subjected to 2000 stretching-releasing cycles at 30% strain along or perpendicular to the channel length direction (fig. S44). The successfully constructed rubbery NIR phototransistors deliver satisfactory performance in terms of semiconductor materials, mechanical stretchability, and optoelectronic properties, compared to other state-of-the-art deformable (intrinsically stretchable or flexible) NIR phototransistors in the literature (table S3).

### Multiplexed rubbery phototransistor array for optoelectronic barcode

**Fig. 5.**
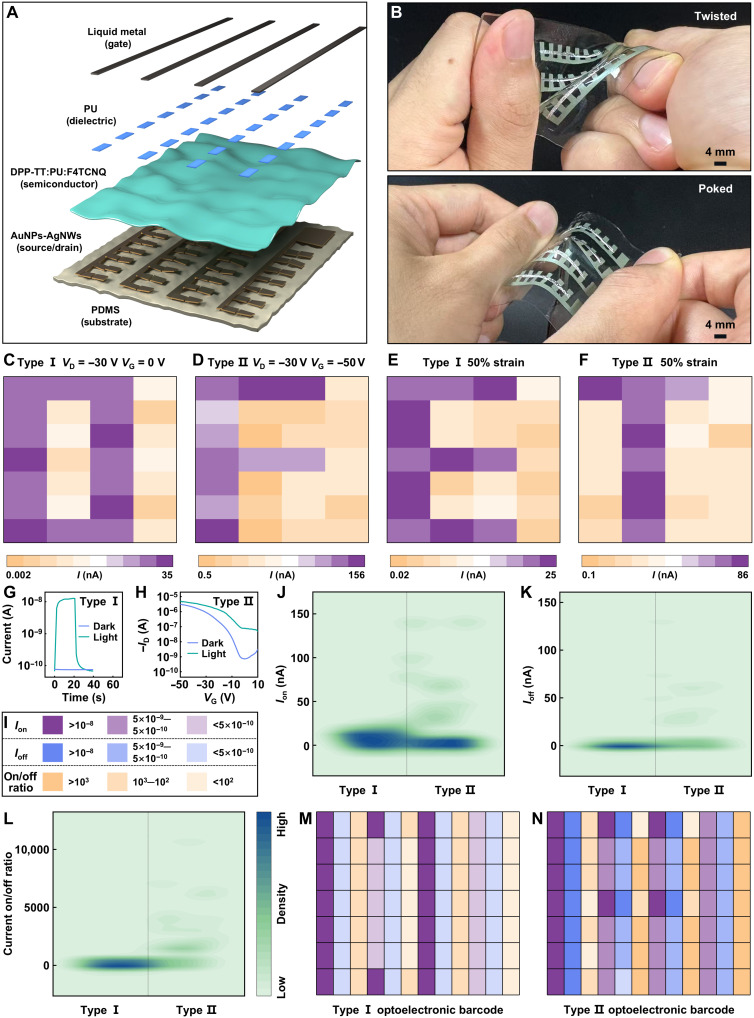
Stretchable 2D optoelectronic barcodes operation on rubbery phototransistor arrays. (**A**) Schematic illustration of the multiplexed rubbery phototransistor array. (**B**) Designed device under twisting and poking deformations. (**C** and **D**) Current mapping of the NIR response from a 4 × 7 rubbery phototransistor array under the illumination of 5.08 mW/cm^2^. (**E** and **F**) The corresponding current mapping of imaging systems stretched at 50% strain. (**G**) The recorded temporal responses of two pixels in fabricated rubbery phototransistor array with/without the NIR illumination of 5.08 mW/cm^2^ in type I array. (**H**) The recorded transfer curves of two pixels in the fabricated rubbery phototransistor array with/without the NIR illumination of 5.08 mW/cm^2^ in type II array. (**I**) Diagram showing the three parameters *I*_on_, *I*_off_, and current on/off ratio defined as purple, blue, and yellow, respectively. A further subdivision of these indices was performed according to their magnitude. (**J** to **L**) Kernel density estimation (KDE)–based heatmap. The summarized *I*_on_ (J), *I*_off_ (K), and current on/off ratio (L) of all pixels in type I and II arrays. In particular, the *I*_on_ and current on/off of pixels located in the obstructed area in type I array are designated as *I*_off_ and 1, respectively. (**M** and **N**) The comprehensive 2D optoelectronic barcodes operation on the rubbery phototransistor type I array (M) and type II array (N).

To confirm the imaging capability of the rubbery phototransistor, we fabricated a 4 × 7 rubbery phototransistor array (fig. S45). The schematic of the exploded view is shown in Fig. 5A. The ultrasensitive phototransistor array exhibits excellent mechanical compliance and ductility even at twisted, poked, and curved states attributed to the inherent stretchability of all its constituent materials (Fig. 5B and fig. S46). To demonstrate the proof-of-concept rubbery image sensor that converts optical patterns into corresponding electrical patterns, the custom-designed masks featuring the letters “O,” “F,” “E,” and “T” are placed between a laser source and phototransistor array, respectively. Noticeably, the fabricated phototransistor array could be operated in two modes, type I and type II, respectively (Fig. 5, C and D). Type I is conducted at *V*_D_ = −30 V and *V*_G_ = 0 V under the illumination of 5.08 mW/cm^2^. A key requirement for high-quality imaging is uniform photoresponse across all pixels under consistent illumination. This uniformity is confirmed by the minimal variation in both dark current and photocurrent, which directly resulted in the accurate capture of the image pattern for “O” (Fig. 5C). Figure 5G depicts the collected data from two representative pixels in the type I array: one located in the illuminated area and the other in the obstructed (dark) area. Type II is measured at *V*_D_ = −30 V and *V*_G_ = −50 V under the illumination of 5.08 mW/cm^2^. Leveraging the high photosensitivity at *V*_G_ = −1 V, we used the corresponding current to plot the NIR imaging pattern. The phototransistor array shows outstanding uniformity in both dark current and photocurrent, yielding a well-defined image pattern for “F” (Fig. 5D). The corresponding signals from two pixels in the type II array (one illuminated and one obstructed) are shown in Fig. 5H. Even when subjected to ~50% strain, the phototransistor array maintained its imaging functionality without notable degradation in dark current or photocurrent. This mechanical robustness enabled the clear formation of the “E” and “T” patterns in the type I and type II arrays, respectively (Fig. 5, E and F), confirming the system’s durability. These results underscore the potential of our multiplexed rubbery phototransistor array for stretchable spatiotemporal imaging.

Highly sensitive capture is facilitated by three clearly distinguishable output indicators: *I*_on_, *I*_off_, and current on/off ratio, which are color coded in purple, blue, and yellow, respectively (Fig. 5I). The multiparameter strategy, using a combination of all *I*_on_, *I*_off_, and the *I*_on_/*I*_off_ ratio, enables the barcoding structure to support a range of security levels, from basic to high assurance, across diverse applications. This dedicated design serves to improve both encoding capacity and hierarchical security at once. Furthermore, *I*_on_ > 10^−8^ A, 5 × 10^−9^ A > *I*_on_ > 5 × 10^−10^ A, and *I*_on_ < 5 × 10^−10^ A are regarded as deep purple, medium purple, and light purple, respectively. Similarly, according to the magnitude of *I*_off_, it is divided into deep blue, medium blue, and light blue. The current on/off ratio is defined as deep yellow (>10^3^), medium yellow (10^2^ to 10^3^), and light yellow (<10^2^). The illumination-dependent electrical curves could subtly manipulate the on/off ratio and current of the different output pixels in the type I and type II arrays, which are measured as optoelectronic signals for the promising application of the optoelectronic barcode. The current on/off ratio, *I*_on_, and *I*_off_ of all pixels in type I and type II arrays are summarized in Fig. 5 (J to L, respectively). Each pixel in the optoelectronic barcode is generated by processing the output signals from three corresponding parameters that populated one by one to form the complete image. Last, the three subsets of type I and type II arrays are integrated into comprehensive 2D optoelectronic barcodes for information processing and optoelectronic encryption (Fig. 5, M and N). Under 50% strain, the NIR imaging systems retain their ability to produce accurate 2D optoelectronic barcodes, demonstrating the mechanical reliability and accuracy of the illumination-dependent electrical behaviors and optoelectronic signals under mechanical deformation (fig. S48). Compared with other encoding methods (table S4), the designed phototransistor array could ensure stable classification of *I*_on_, *I*_off_, and current on/off ratio despite variations in illumination. Moreover, the highly conductive and mechanically stretchable nature of the liquid metal facilitates a uniform gate potential, resulting in negligible gate bias drift (fig. S50). The demonstration on stretchable 2D barcode promotes next-generation rubbery electronics with desired reading accuracy and encoding capacity. Leveraging the unique advantages of rubbery phototransistors within optoelectronic interconnected structures enables precise multichannel signal output, thus advancing the development of skin-like sophisticated multifunctional devices.

## DISCUSSION

In summary, we have developed a multiplexed, rubbery NIR phototransistor array via a scalable air/water interfacial self-assembly of a ternary system (DPP-TT, F4TCNQ, and PU). The resulting DFP nanofilm achieves high-sensitivity NIR detection through the synergistic combination of p-type chemical doping and a spatially nanoconfined morphology. Noticeably, the percolating nanoconfined nanoweb structure yields a unique combination of exceptional mechanical stretchability and high charge carrier mobility. The developed rubbery NIR phototransistor arrays demonstrate precise NIR pattern detection and accurate image sensing, even when subjected to 50% strain. The imaging systems with multichannel signal outputs achieve precise manipulation of optoelectrical transformation, which facilitates the realization of optoelectronic barcodes for information recording and encryption. This work establishes a robust and scalable platform for high-performance rubbery optoelectronics.

## MATERIALS AND METHODS

### Materials

AgNW (~99.5%) solution was purchased from ACS Material. Chloroform (>99%), 1,2-dichlorobenzene (>99%), anhydrous tetrahydrofuran (>99%), anhydrous ammonia (28%), gold chloride trihydrate (>99.9%), PU, polystyrene-block-poly(ethylene-ran-butylene)-block-polystyrene (SEBS), liquid metal, and F4TCNQ were purchased from Sigma-Aldrich. Poly{2,2′-[(2,5-bis(2-octyldodecyl)-3,6-dioxo-2,3,5,6-tetrahydropyrrolo[3,4-c]pyrrole-1,4-diyl)]dithiophene-5,5′-diyl-alt-2,2′-bithiophene-5,5′-diyl} (DPP4T), poly(3-hexylthiophene-2,5-diyl) (P3HT), poly[2,5-bis(2-decyltetradecyl)pyrrolo[3,4-c]pyrrole-1,4(2*H*,5*H*)-dione-alt-5,5′-di(thiophen-2-yl)-2,2′-(*E*)-2-(2-(thiophen-2-yl)vinyl)thiophene] (PDVT-10), and DPP-TT were received from Hangzhou Order Science & Technology Co. Ltd. PDMS (Sylgard 184) was sourced from Dow Corning.

### Methods

#### Preparation of the air/water interface–assembled DPP-TT rubbery semiconducting nanofilms

Semiconducting polymer DPP-TT was fully dissolved in its designated mixed solvent (chloroform and 1,2-dichlorobenzene with volume ratio of 9:1) with concentrations of 5.0 mg ml^−1^ at 60°C under continuous stirring to prepare homogeneous solutions. The elastomer PU was first dissolved in tetrahydrofuran (25 mg ml^−1^) and then blended with the DPP-TT solution at specific volume ratios. Homogeneous mixtures were achieved after vigorous stirring for at least 1 hour. By depositing a controlled volume of the blended polymer solution onto the air/water interface, subsequently large-area, continuous, and freestanding rubbery semiconducting nanofilms were formed. The facile transfer of these nanofilms onto diverse substrates, including silicon wafers, glass, and PDMS, enabled their subsequent characterization and integration into functional devices.

#### Preparation of the highly doped DPP-TT semiconducting nanofilms

Molecular dopant F4TCNQ was fully dissolved in chloroform with concentrations of 0.5 mg ml^−1^ at room temperature under continuous stirring. The DPP-TT polymer solution was blended at specific ratios, followed by vigorous stirring to achieve homogeneous mixtures. The polymer films were deposited on the cleaned substrates by spin coating the resulted highly doped polymer solution at 3000 rpm for 60 s and then annealed at 60°C for at least 1 hour.

#### Preparation of the rubbery doping nanofilm

Following the addition of the dopant to the DPP-PU polymer solution at specific ratios, the mixture was heated (60°C for 1 hour) to ensure homogeneity and then deposited onto the air/water interface, resulting in a uniform rubbery doping nanofilm.

#### Fabrication of spin-coated film-based transistor

AuNPs-AgNWs/PDMS-based stretchable electrodes served as the source and drain contacts, which were micropatterned using the reported procedure (*42*). The semiconductor layer was subsequently spin coated from polymer solution onto these electrodes. The obtained semiconductor films were annealed at 60°C for 1 hour to remove residual solvent. Then, a dielectric layer was fabricated by spin coating a PU solution (75 mg ml^−1^) onto the channel layer at 3000 rpm for 60 s, followed by annealing at 80°C for 1 hour. Last, a liquid metal gate electrode was patterned onto the PU dielectric layer through a shadow mask using a doctor-blading technique to complete the device fabrication.

#### Preparation of the rubbery transistor based on air/water interface–assembled semiconducting nanofilm

The assembled DPP-TT rubbery nanofilm was precisely transferred and laminated onto the stretchable electrodes. Subsequent device completion involved a natural air-drying period overnight with a secondary drying step at 60°C for 1 hour, which ensured the thorough elimination of residual water. Thereafter, the gate dielectric and gate electrode were deposited according to the previously described fabrication protocol for spin-coated film-based transistors.

#### Measurements and characterizations

The electrical transport and optoelectronic characteristics were tested by using a semiconductor analyzer (Keithley 4200A-SCS) coupled with a probe station (H100, Signatone). GIWAXS patterns were performed on an Anton Paar SAXS point 2.0 system. The diffraction signal was obtained using a 2D Hybrid Photon-Counting detector, in which all tests were conducted under vacuum conditions to reduce air scattering and prevent beam-induced sample damage. XPS analysis was carried on the Thermo Fisher Scientific K-Alpha system with monochromatic Al Kα x-rays (1486.6 eV). UV-vis-NIR absorption was performed on Lambda 1050+, using a blank quartz glass substrate as the reference baseline. Atomic force microscopy and Dimension Icon were used to analyze the morphology of the assembled nanofilm. For irradiation, we used 808-nm lasers as the light sources (Nanjing Latron Laser Company, China). UPS analysis was conducted on Thermo ESCALAB 250XI.

#### Optoelectronic imaging experimental procedures

An imaging system integrated with phototransistors was constructed to verify the feasibility of our stretchable phototransistors with high detectivity. Under the irradiation of NIR light source with the wavelength of 808 nm, optical patterns projected on imaging systems can be identified through the phototransistor arrays. In the imaging experiment, lab-made masks with different patterns are placed on the platform between the rubbery transistor array and incident laser, and this platform can be moved linearly in the *X*-*Y* platform. The phototransistor device acts as an imaging sensor to record the position-dependent real-time current variation due to light illumination through the patterned mask. Last, imaging results for objects were drawn through converting photocurrent data into the corresponding high-resolution patterned image.

## Supplementary Materials

### This PDF file includes:

Supplementary Text Figs. S1 to S51 Tables S1 to S4 References
