# Supplementary material for: A rubbery semiconducting heterojunction film for fully rubbery multiplexed near-infrared phototransistor arrays
Source: Sci Adv. 2026 May 27;12(22):eaee2602. doi: 10.1126/sciadv.aee2602 (PMC13215191; doi:10.1126/sciadv.aee2602)
Supplement: Supplementary file 1 — Supplementary Text Figs. S1 to S51 Tables S1 to S4 References [file sciadv.aee2602_sm.pdf]

Science Advances
AAAS

Supplementary Materials for

A rubbery semiconducting heterojunction film for fully rubbery multiplexed near-infrared phototransistor arrays

Yu-Dong Zhao *et al.*

Corresponding author: Jing Qiao, qiaojing0702@seu.edu.cn; Ying-Shi Guan, gyshi412@seu.edu.cn

Sci. Adv. **12**, eaee2602 (2026)
DOI: 10.1126/sciadv.aee2602

This PDF file includes:

Supplementary Text
Figs. S1 to S51
Tables S1 to S4
References

Supplementary Text

Calculation of field-effect mobility.

The field-effect mobility (μ_{FE}) was calculated by fitting the plot of the linear regime of the square root of the drain current ($\sqrt{I_{DS}}$) versus gate voltage (V_{GS}) curve based on the following equations:

$$I_{DS} = \frac{WC_i\mu_{FE}}{2L}(V_{GS} - V_{TH})^2 \tag{S1}$$

$$\sqrt{I_{DS}} = \sqrt{\frac{WC_i\mu_{FE}}{2L}}(V_{GS} - V_{TH}) = \sqrt{\frac{WC_i\mu_{FE}}{2L}}V_{GS} - \sqrt{\frac{WC_i\mu_{FE}}{2L}}V_{TH} \tag{S2}$$

$$Slope = \sqrt{\frac{WC_i\mu_{FE}}{2L}} \tag{S3}$$

$$\mu_{FE} = \frac{2L}{WC_i}(Slope)^2 \tag{S4}$$

where L and W are the length and width of the channel. The C_i is the gate insulator capacitance per unit area. I_{DS} is the drain current, and μ_{FE} is the field-effect mobility, respectively. V_{GS} is the gate voltage.

Density functional theory calculations.

The theoretical calculations are conducted using the Gaussian 09 software packages. The geometry optimizations of the individual molecules were optimized at the B3LYP/6-31+G(d,p) level, long alkyl chains were instead by methyl to simplify calculation. The ESP was obtained by a wavefunction analysis tool Multiwfn (43).

Calculation of paracrystalline disorder parameter *g*.

To quantify the microstructural disorder of DPP-TT thin films, we calculate the paracrystalline disorder parameter for the lamellar stacking and π-π packing along these two directions, which is defined according to the following equation:

$$g = \sqrt{\frac{\Delta q}{2\pi q_0}}$$

where Δq is the FWHM of a diffraction peak and q_0 is the center position of the same peak (44).

The typical parameters of phototransistors are defined according to the following equations:

$$\text{Photosensitivity } (P) = \frac{I_{light} - I_{dark}}{I_{dark}}$$

$$\text{Photoresponsivity } (R) = \frac{I_{light} - I_{dark}}{E_e\, S}$$

$$\text{Detectivity } (D^*) = R\sqrt{\frac{S}{2\, e\, I_{dark}}}$$

$$\text{External quantum efficiency } (EQE) = \frac{R\, h\, c}{\lambda\, e}$$

where I_{light} and I_{dark} refer to the channel current under illumination and in the dark. E_e is the incident light power density. S represents the active area, e represents the elemental electron, and hc/λ represents the photon energy.

The comparison of contact resistance (R_C) between AuNPs–AgNWs or AgNWs and semiconductor DPP-TT.

The contact resistance between AuNPs–AgNWs or AgNWs and semiconductor DPP-TT are extracted through the transfer length method (TLM), and the results are displayed in Fig. S8. The total device resistance R_{ON} is given by the channel resistance R_{CH} (a quantity which is proportional to the channel length) and the contact resistance R_C, which was determined according to the following method (21).

$$R_{\mathrm{ON}}\ (\mathrm{L}) = R_{\mathrm{CH}}\ (\mathrm{L}) + R_C$$

The R_{ON} with a different channel length was characterized as V_{GS} of -40 V and V_{DS} of -5 V ($V_{DS} << V_{GS}-V_{TH}$) (Fig. S8A, B). Significantly, the width-normalized R_C ($R_C \cdot W$) can be extracted from the y-intercept of the extrapolated plot of the width-normalized R_{ON} ($R_{ON} \cdot W$) versus channel length. The fitted plot in Fig. S8C sufficiently confirms the decrease in R_C and optimized interface contact.

Gate bias dependence of photosensitivity, responsivity, and detectivity.

The photosensitivity realizes the maximum at around V_G = -1 V, ascribed to the fact that the constructed rubbery NIR phototransistors are in the off-state when V_G = -1 V in the dark, but switch to the on-state upon illumination due to the positively shifted V_{th}. Responsivity achieves the maximum in the range of -35 V to -50 V owing to the generated larger photocurrent under progressively higher gate bias, which facilitates more effective carrier dissociation and hole transport in the semiconductor layer. The variation of detectivity is dictated by the trade-off between enhancing photocurrent generation (responsivity) and suppressing all noise mechanisms.

The specific output indicators and operating conditions of the fabricated NIR imaging system and optoelectronic barcode.

The four devices operating under distinct conditions are used to illustrate their respective working principles (Figs. S48 and S49). The type I mode is conducted at constant V_D of -30 V and V_G of 0 V while the type II mode is measured by sweeping the input voltage from 10 V to -50 V with a fixed V_D of -30 V. Devices 2 and 4 are exposed to NIR light (5.08 mW/cm^2), whereas devices 1 and 3 are kept in the dark (Fig. S48A, B).

Fig. S48C, D indicates the $I_{measured}$ for current mapping (Fig. 5C-F), respectively. For type I mode, the $I_{measured}$ of device 2 refers to the largest photocurrent whereas $I_{measured}$ of device 1 shows a nearly linear response. For type II mode, the $I_{measured}$ of both devices 3 and 4 are extracted from the V_G of -1 V, based on the achieved maximum photosensitivity at around V_G = -1 V (Fig. S48E).

Fig. S48F-I points out the $I_{measured}$ for optoelectronic barcode (Fig. 5G-N), respectively. The color coding according to Fig. 5I is also presented in the corresponding device. For device 1, the I_{on} and I_{off} are defined as equal, thus yielding a fixed current on/off ratio of 1, owing to the nearly linear current response observed in the dark. For device 2, the I_{on} refers to the largest photocurrent and I_{off} is stable current measured after the light is switched off. The on/off ratio is calculated from the aforementioned values of I_{on} and I_{off}. For devices 3 and 4, the I_{off} refers to the smallest photocurrent and I_{on} is extracted from the V_G of -1 V, the on/off ratio is calculated from the I_{off} and the largest photocurrent. From a signal-recognition standpoint, conflating I_{on} with the simple maximum photocurrent is inadvisable, as it compromises the discrimination of distinct signal levels (Fig. S49). The optimal distinguishability achieved at -1 V is attributed to its maximized current differential, which sharpens the separation between defined states and minimizes informational overlap.

Validation of imaging results reliability.

The observed negligible change in the resistance of liquid metal gate electrode during dynamic stretching (more than 6000 cycles at 50% strain) effectively eliminates crosstalk stemming from gate voltage non-uniformity (Fig. S50). The recorded transfer characteristics of the selected pixel and adjacent pixels confirm the reliability of the imaging results even under mechanical deformation (Fig. S51).

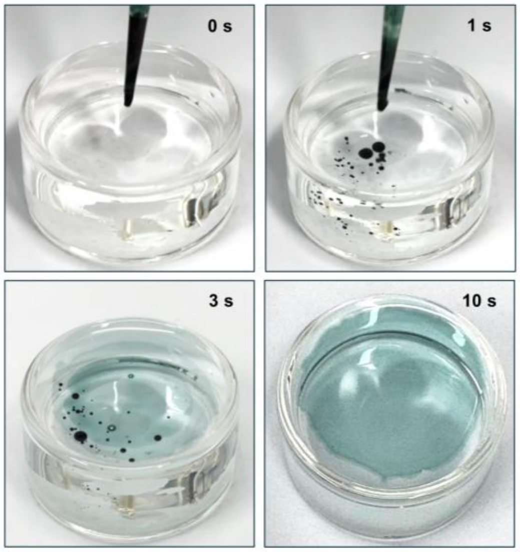

Fig. S1. Sequential images of the self-assembly process during doping. The optical images of the rubbery doping DPP-TT nanofilm formed on the water surface.

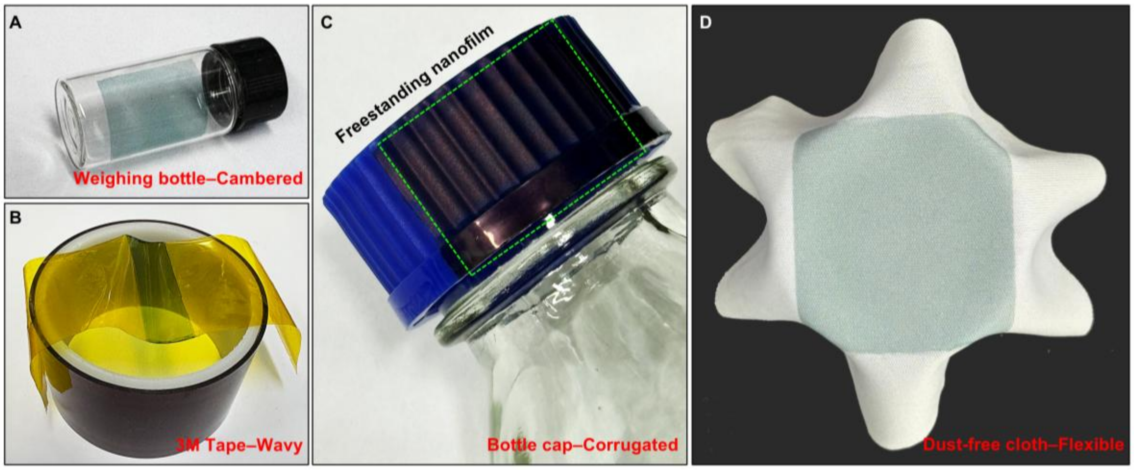

Fig. S2. Transfer process of DFP nanofilm. Rubbery doping DPP-TT nanofilm transferred to different substrates, including weighing bottle (**A**), smooth 3M tape(**B**), bottle cap (**C**) and dust-free cloth (**D**).

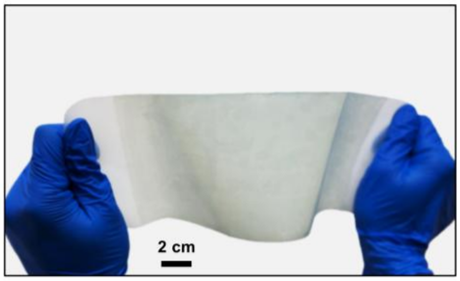

Fig. S3. The scalability of rubbery semiconductors by air/water interfacial self-assembly method. The optical image of the large-area freestanding rubbery semiconducting nanofilms with 25×10 cm^2 area on PTFE substrate.

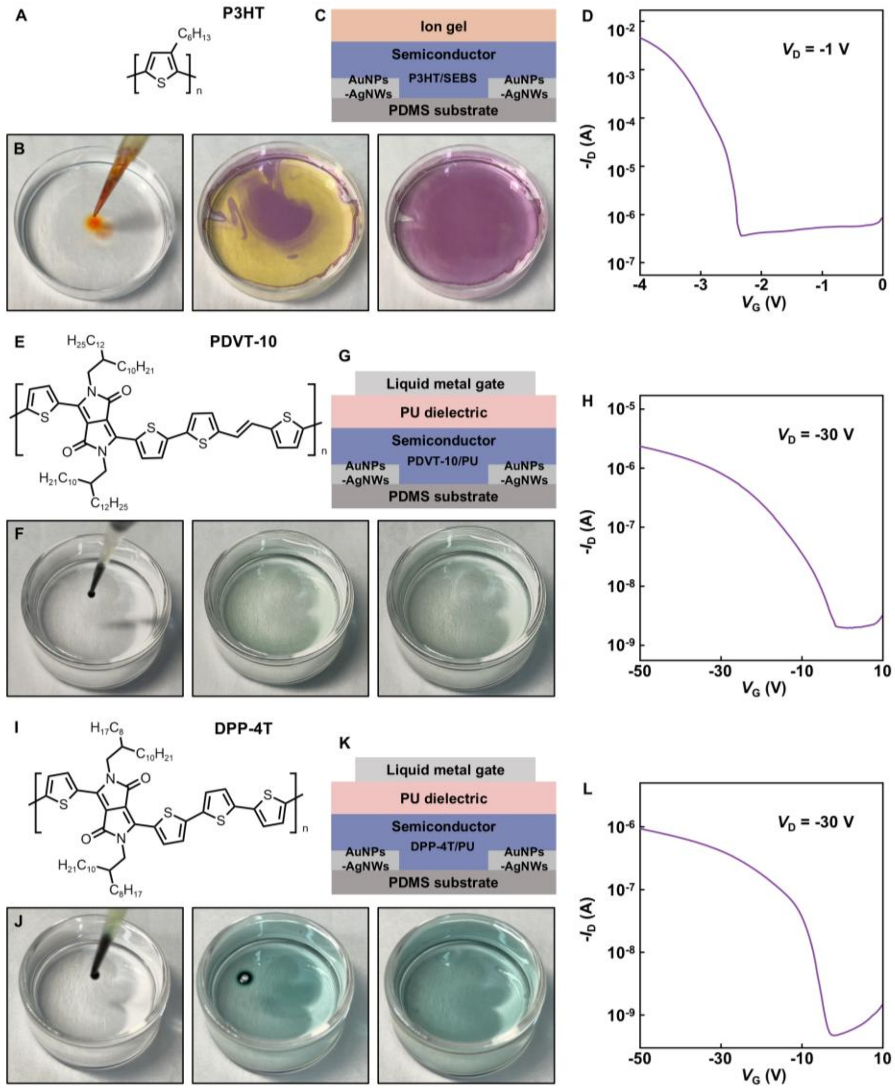

Fig. S4. General applicability of air/water interfacial self-assembly method on polymeric semiconductor film-based transistors. (**A**, **E**, **I**) Chemical structures of polymeric semiconductors P3HT (**A**), PDVT-10 (**E**), and DPP-4T (**I**). (**B**, **F**, **J**) The optical images of air/water interfacial self-assembly process correspond to P3HT (**B**), PDVT-10 (**F**), and DPP-4T (**J**), respectively. (**C**, **G**, **K**) The device configurations of polymeric semiconductor film-based transistors correspond to P3HT (**C**), PDVT-10 (**G**), and DPP-4T (**K**), respectively. (**D**, **H**, **L**) The representative transfer curves correspond to P3HT (**D**), PDVT-10 (**H**), and DPP-4T (**L**), respectively.

The dynamic self-assembly mechanism based on the air/water interfacial process is elucidated as follows. The deposition of a low-surface-tension liquid onto a high-surface-tension creates a surface tension gradient which induces a Marangoni flow that propelling the lower-tension liquid toward regions of higher tension (45). During solvent evaporation, semiconductor molecules spontaneously organize into well-arrangement nanostructures at the air/water interface through

intermolecular forces, notably π-π stacking and hydrophobic interactions (46). The hydrophobic characteristic of the semiconducting polymers drives the polymer chains to pack densely into an edge-on stacking for minimizing the contact of polymer chains with water. Therefore, the evaporation-driven self-assembly of polymer chains on water is vital for the formation of ordered structure, which is agreed with the obtained idea transfer curves.

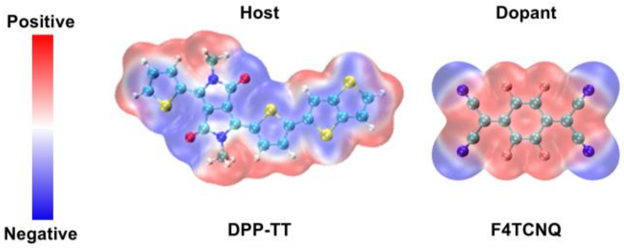

Fig. S5. The surface electrostatic potential maps of DPP-TT and F4TCNQ.

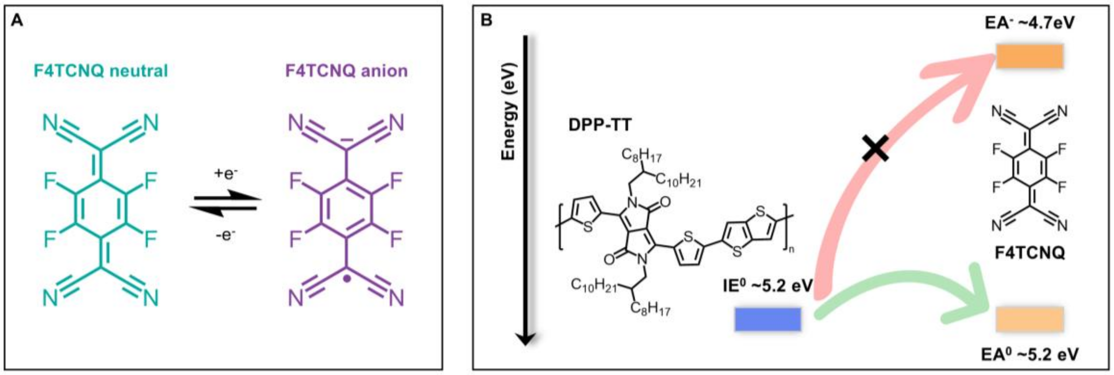

Fig. S6. Energy diagram depicting the formation of dopant anions. (**A**) Diagram showing the F4TCNQ reduction yields a stable aromatic radical anion. (**B**) Green (red) arrows indicate where electron transfer from the polymer to the dopant is observed (absent). The IE^0 of the semiconducting polymer DPP-TT as well as EA^0 and EA^- of the dopant F4TCNQ were referenced the literature (32, 47).

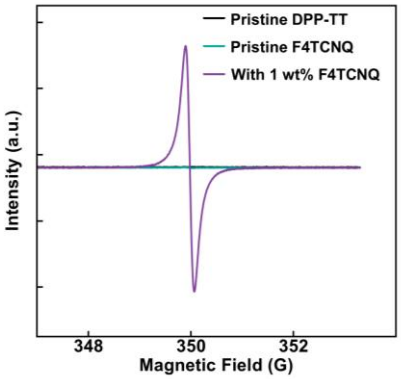

Fig. S7. Characterization of charge transfer nature between DPP-TT and F4TCNQ. EPR spectra of pristine DPP-TT, pristine F4TCNQ and doped DPP-TT with 1 wt% F4TCNQ.

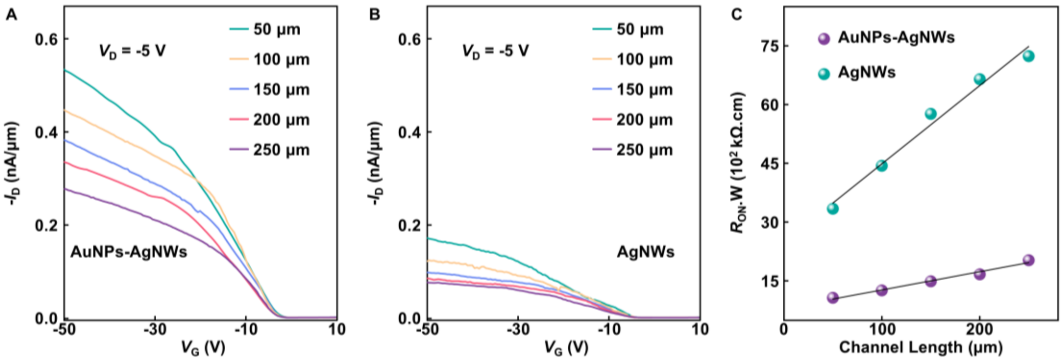

Fig. S8. Contact resistance measurements. (**A**, **B**) Transfer characteristic of pristine DPP-TT nanofilms–transistors based on AuNPs–AgNWs (**A**) and AgNWs (**B**) worked as source–drain electrodes, respectively. (**C**) Resistance changes of OFETs with AuNPs–AgNWs or AgNWs electrode as a function of channel length to compare contact resistance.

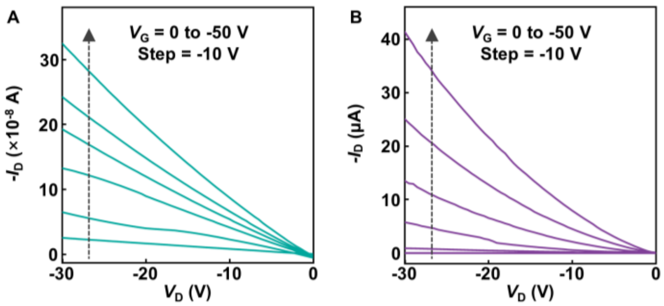

Fig. S9. Output curves. Representative output characteristics of the thin film transistors based on pristine DPP-TT (**A**) and doped DPP-TT (2 wt% F4TCNQ) (**B**).

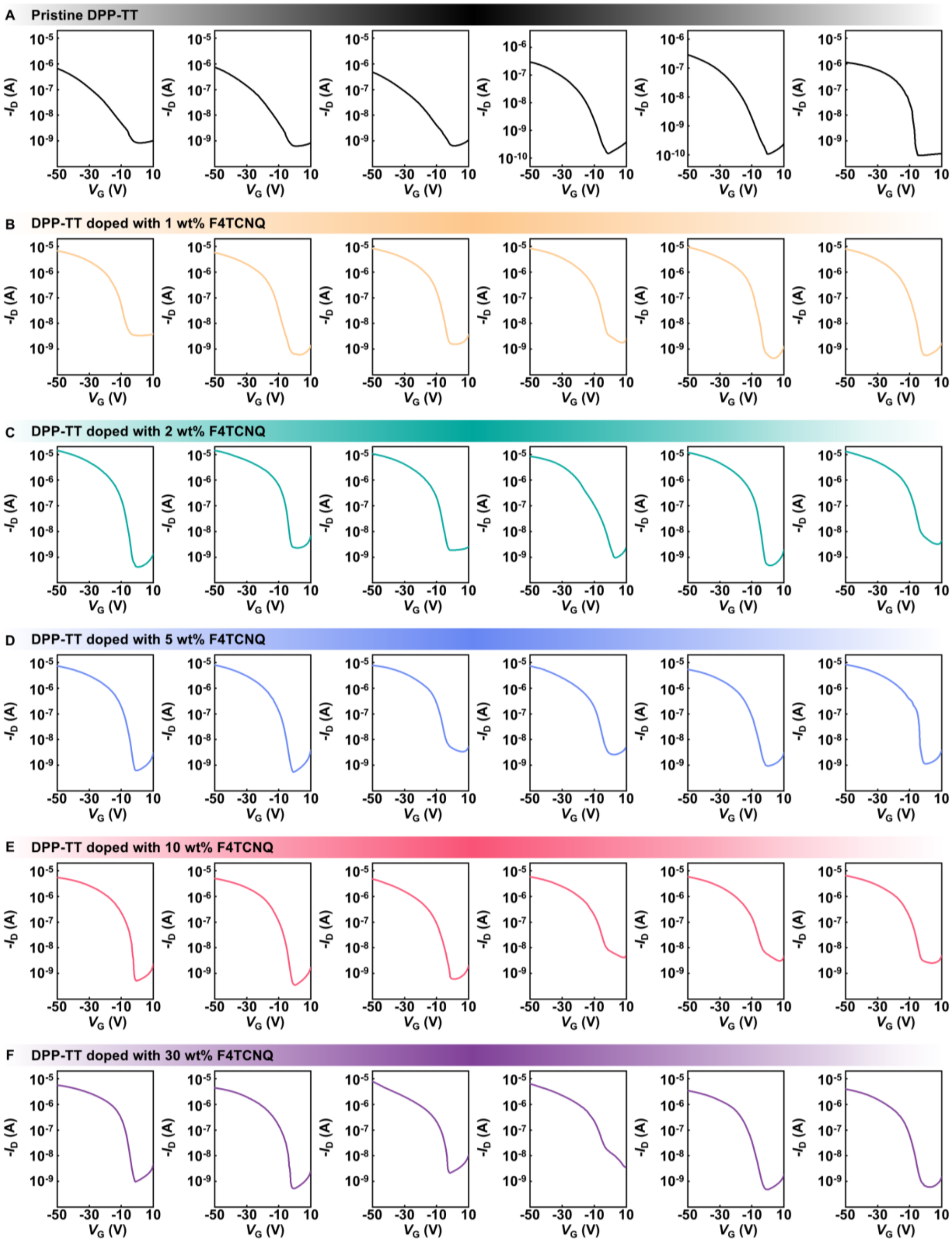

Fig. S10. Transfer curves. Representative transfer characteristics of the thin film transistors based on pristine DPP-TT (**A**) and doped DPP-TT with 1 wt% F4TCNQ (**B**), 2 wt% F4TCNQ (**C**), 5 wt% F4TCNQ (**D**), 10 wt% F4TCNQ (**E**), 30 wt% F4TCNQ (**F**).

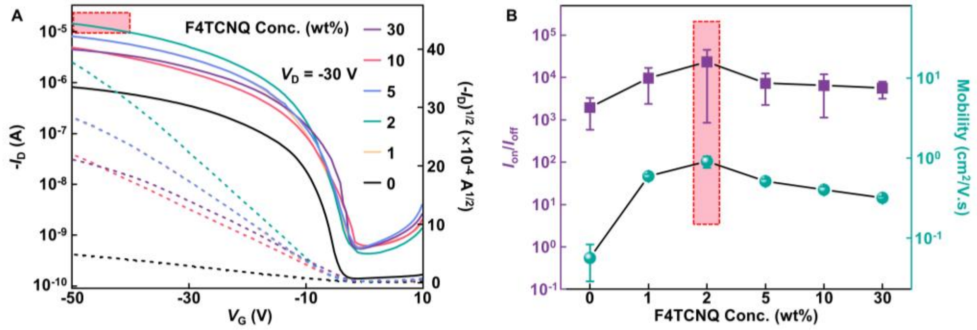

Fig. S11. Electronic performance of the pristine DPP-TT and blended nanofilms. (**A**) Representative transfer characteristics of the thin film transistors based on pristine DPP-TT and doped DPP-TT with 1 wt%, 2 wt%, 5 wt%, 10 wt% and 30 wt% F4TCNQ, respectively. (**B**) The current on/off ratio and charge carrier mobility depending on F4TCNQ concentration.

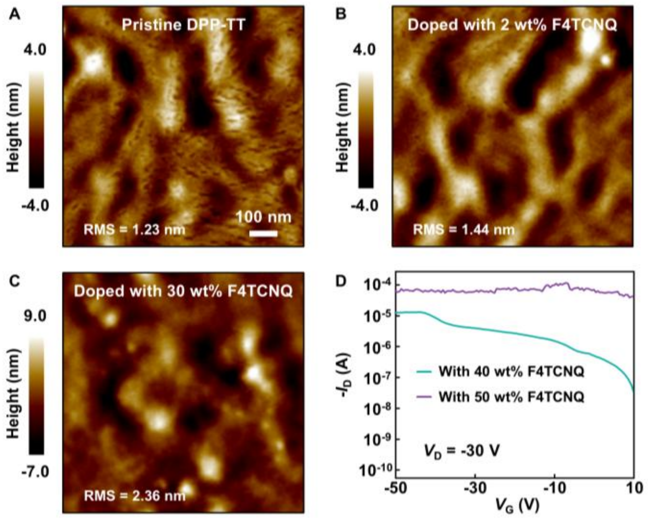

Fig. S12. Analysis of the morphology characterization and electronic performance of heavily doped thin films. (**A**-**C**) AFM height images of the pristine (**A**) and doped DPP-TT nanofilms with 2 wt% (**B**) F4TCNQ and 30 wt% (**C**) F4TCNQ. (**D**) Transfer curves of the thin film transistors based on heavily doped DPP-TT nanofilms (40 wt% and 50 wt% F4TCNQ).

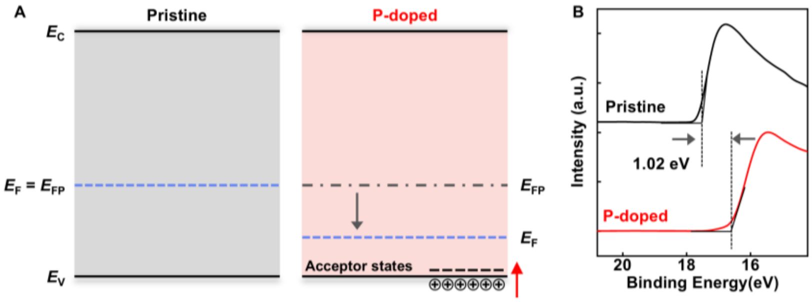

Fig. S13. Understanding of the p-doped effects on the organic semiconductors. (**A**) Energy band diagram for pristine and p-doped semiconductors. Red arrow near acceptor states indicates electron activation leading to dopant ionization, hence generating extrinsic mobile holes in the valence band. E_{FP} represents pristine Fermi level. (**B**) UPS spectra of pristine DPP-TT and doped DPP-TT with 2 wt% F4TCNQ.

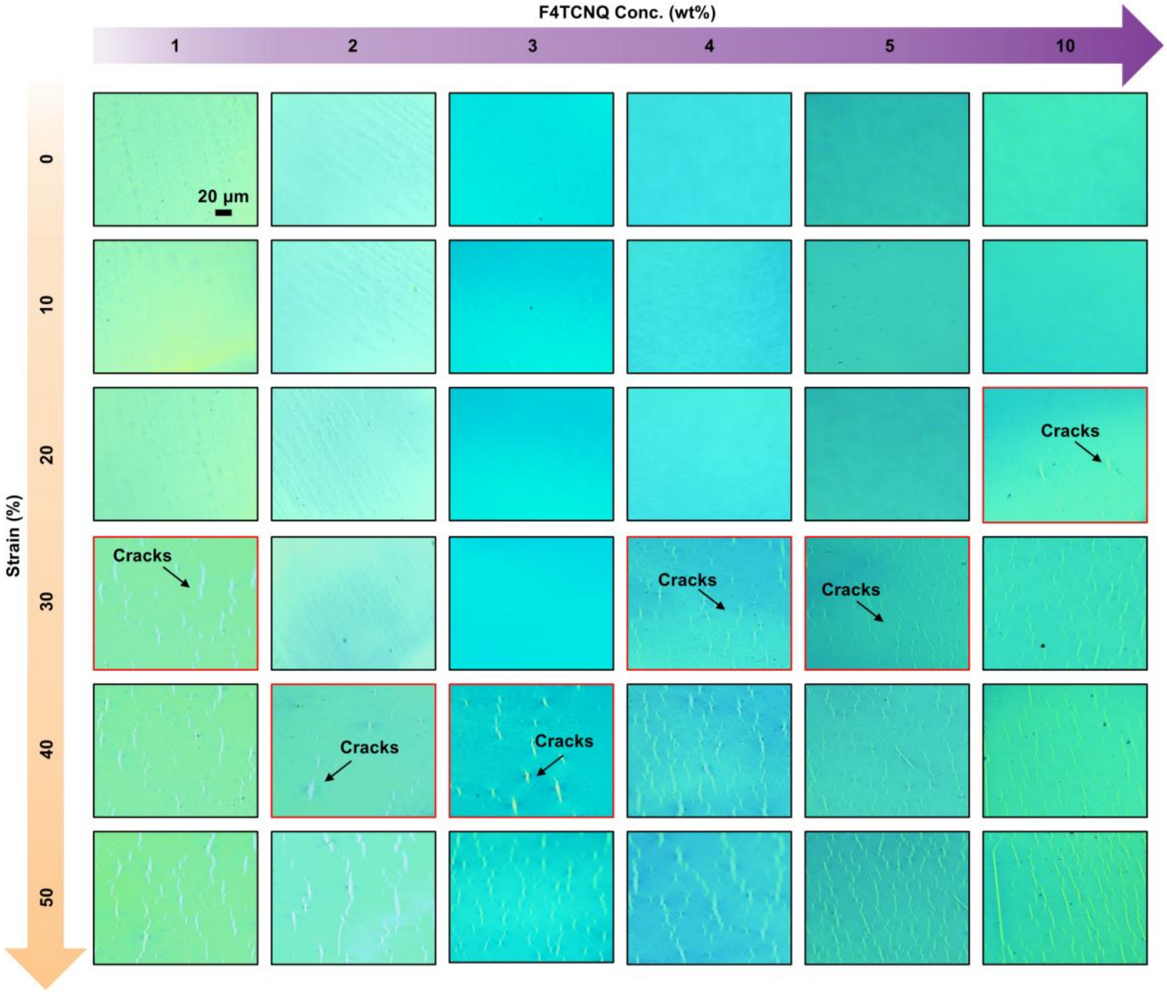

Fig. S14. The optical images of the blended DPP-TT nanofilms with various F4TCNQ ratios (1 wt%, 2 wt%, 3 wt%, 4 wt%, 5 wt% and 10 wt%) stretched at 0%, 10%, 20%, 30%, 40% and 50% strain. The images for the 2 wt% F4TCNQ sample at 0% and 30% strain are intentionally reused from Fig. 1F in the main text to maintain logical consistency and facilitate direct comparison across figures.

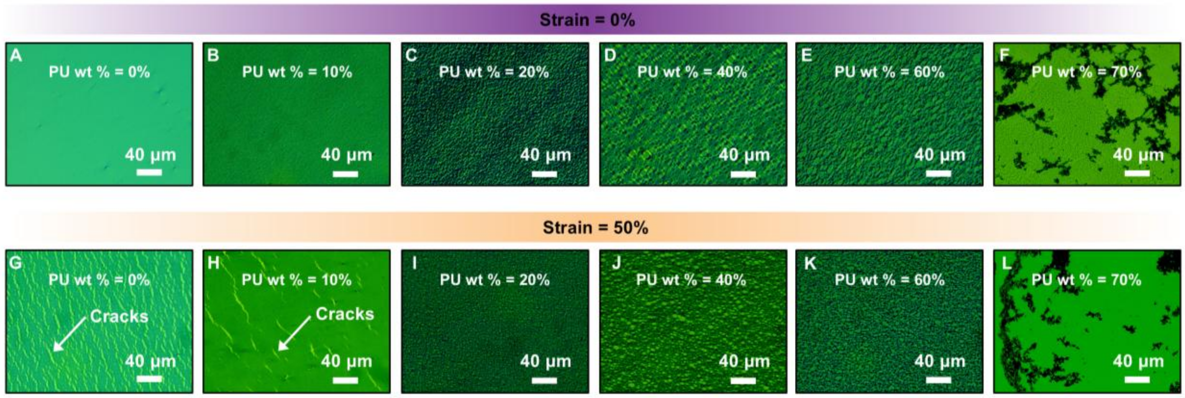

Fig. S15. Phase separation in DPP-TT/PU rubbery semiconducting nanofilms. (**A** to **F**) The optical images of the DPP-TT/PU rubbery semiconducting nanofilms with varying PU percentages at 0% strain. (**G** to **L**) Corresponding optical images of DPP-TT/PU rubbery semiconducting nanofilms stretched at 50% strain. The image in (C) and its stretched counterpart in (I) are intentionally reused from Fig. 1G in the main text to facilitate direct comparison and maintain logical flow across figures.

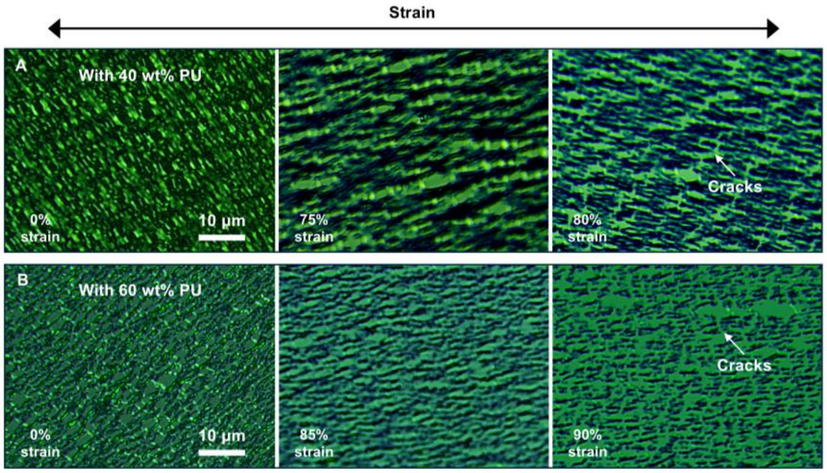

Fig. S16. Optical images of the blended DPP-TT nanofilms with 40 wt% and 60 wt% PU under strain. (**A**) The optical images of the blended DPP-TT nanofilms with 40 wt% PU stretched at 0%, 75% and 80% strain, respectively. (**B**) The optical images of the blended DPP-TT nanofilms with 60 wt% PU stretched at 0%, 85% and 90% strain, respectively.

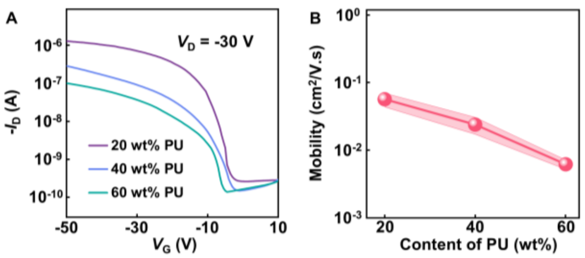

Fig. S17. The air/water assembled freestanding rubbery nanofilms with different percentages of PU. (**A**) Representative transfer curves of transistors based on the rubbery nanofilms with 20 wt%, 40 wt% and 60 wt% PU. (**B**) The corresponding mobilities of the rubbery nanofilms with different percentages of PU.

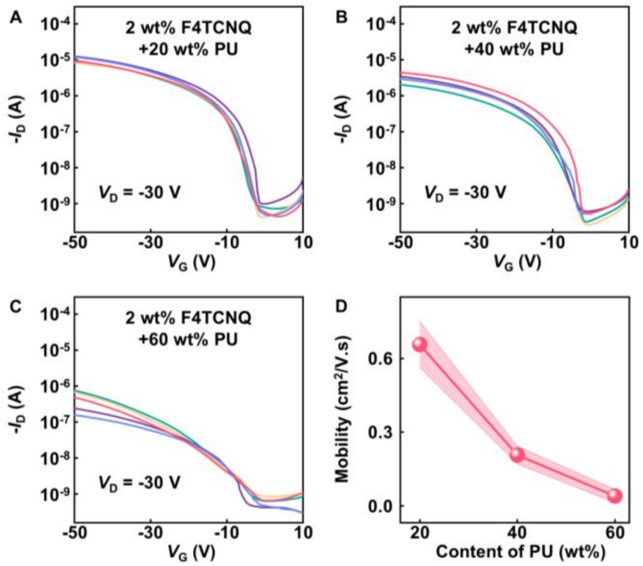

Fig. S18. The air/water assembled freestanding rubbery doping nanofilms with different percentages of PU. Representative transfer curves of transistors based on the rubbery doping nanofilm with 20 wt% PU (**A**), 40 wt% PU (**B**) and 60 wt% PU (**C**). (**D**) The corresponding mobilities of the rubbery doping nanofilms with different percentages of PU.

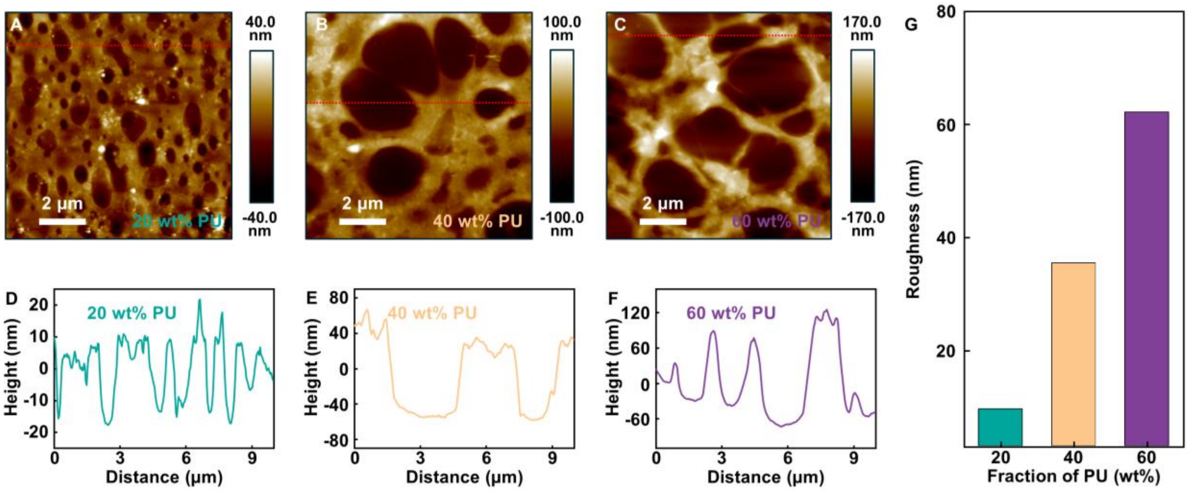

Fig. S19. Morphology of the rubbery semiconducting nanofilms with various weight ratios. (**A** to **C**) AFM height images with the PU contents of 20 wt% (**A**), 40 wt% (**B**), and 60 wt% (**C**). (**D** to **F**) The corresponding height profiles of rubbery semiconducting nanofilms with 20 wt% PU (**D**), 40 wt% PU (**E**), and 60 wt% PU (**F**). (**G**) Corresponding summary of surface roughness. The image in (A) and (C) are intentionally reused from Fig. 2H and 2A in the main text to facilitate direct comparison and maintain logical consistency across figures.

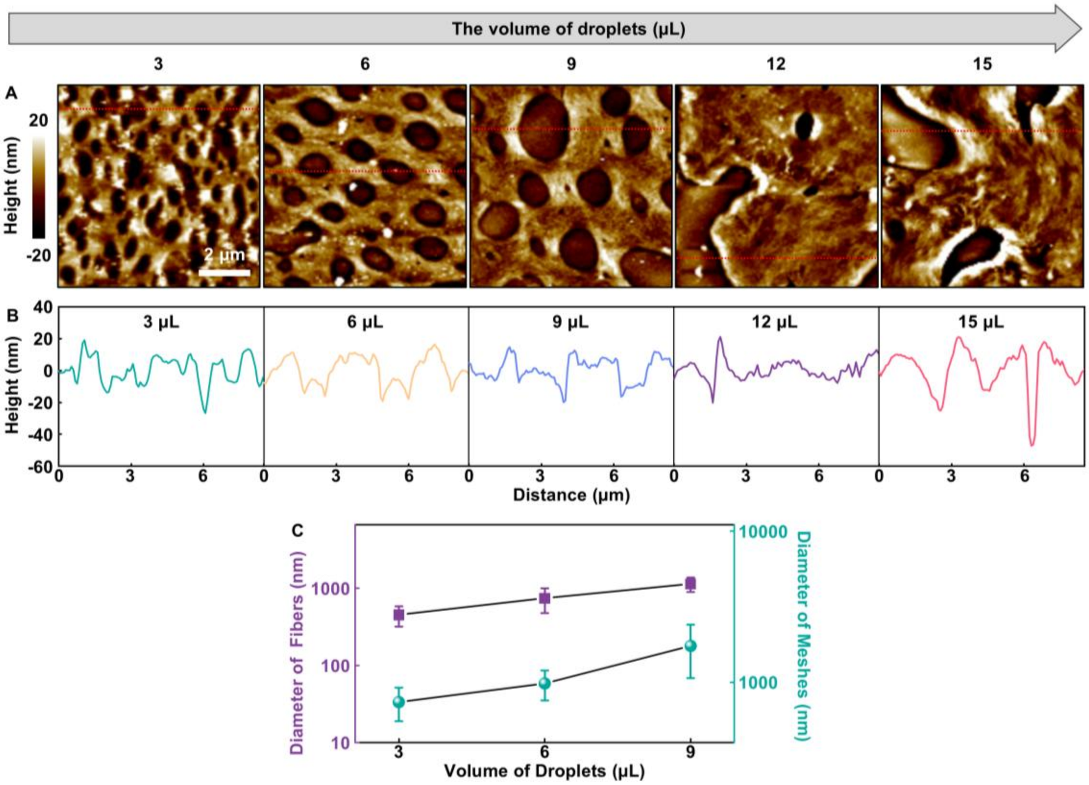

Fig. S20. The influence of the volume of introduced solution per addition on microscopic morphology. (**A**) AFM height images of the rubbery semiconducting nanofilms with 20 wt% PU fabricated from different volumes of introduced solution per addition. (**B**) The corresponding height profiles. (**C**) Diameter variations of fibers and meshes with increasing the volume of introduced solution per addition from 3 μL to 9 μL.

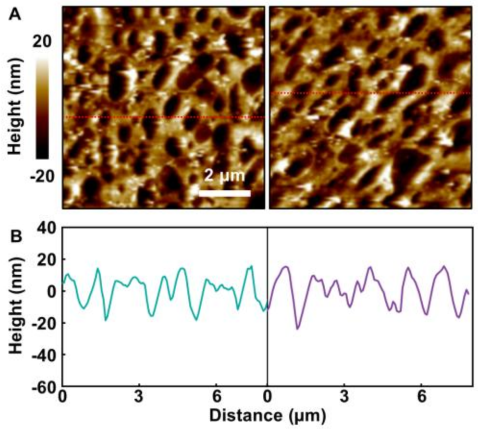

Fig. S21. Two samples that self-assembled under the same conditions of 20 wt% PU and 3 μL introduced solution per addition. (**A**) AFM height images. (**B**) The corresponding height profiles.

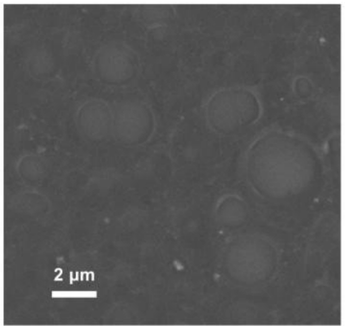

Fig. S22. The SEM image of the rubbery semiconducting nanofilm (20 wt% PU) acquired without sputter-coating conductive gold layer to preserve the native surface topography.

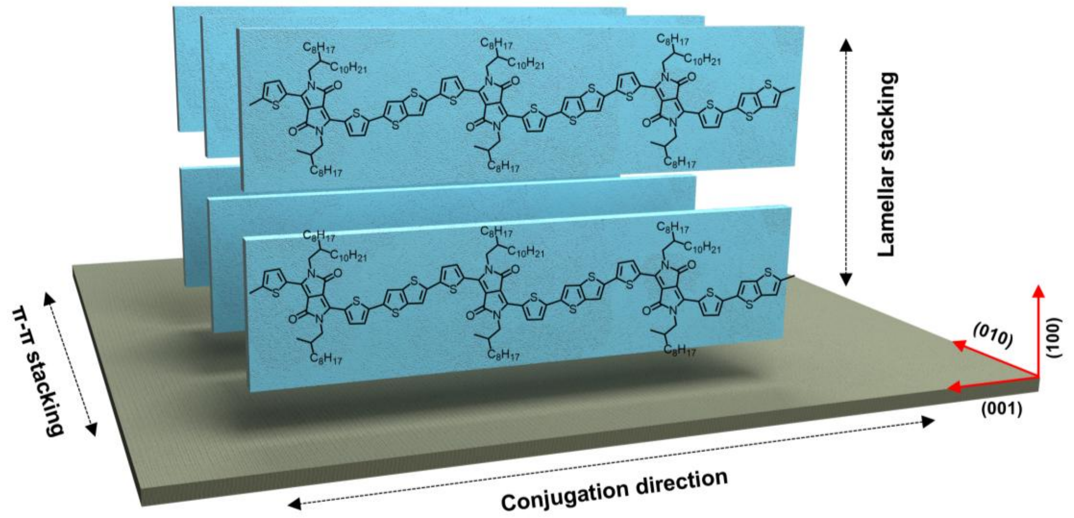

Fig. S23. The schematic illustration of the packing configuration in the DPP-TT nanofilm.

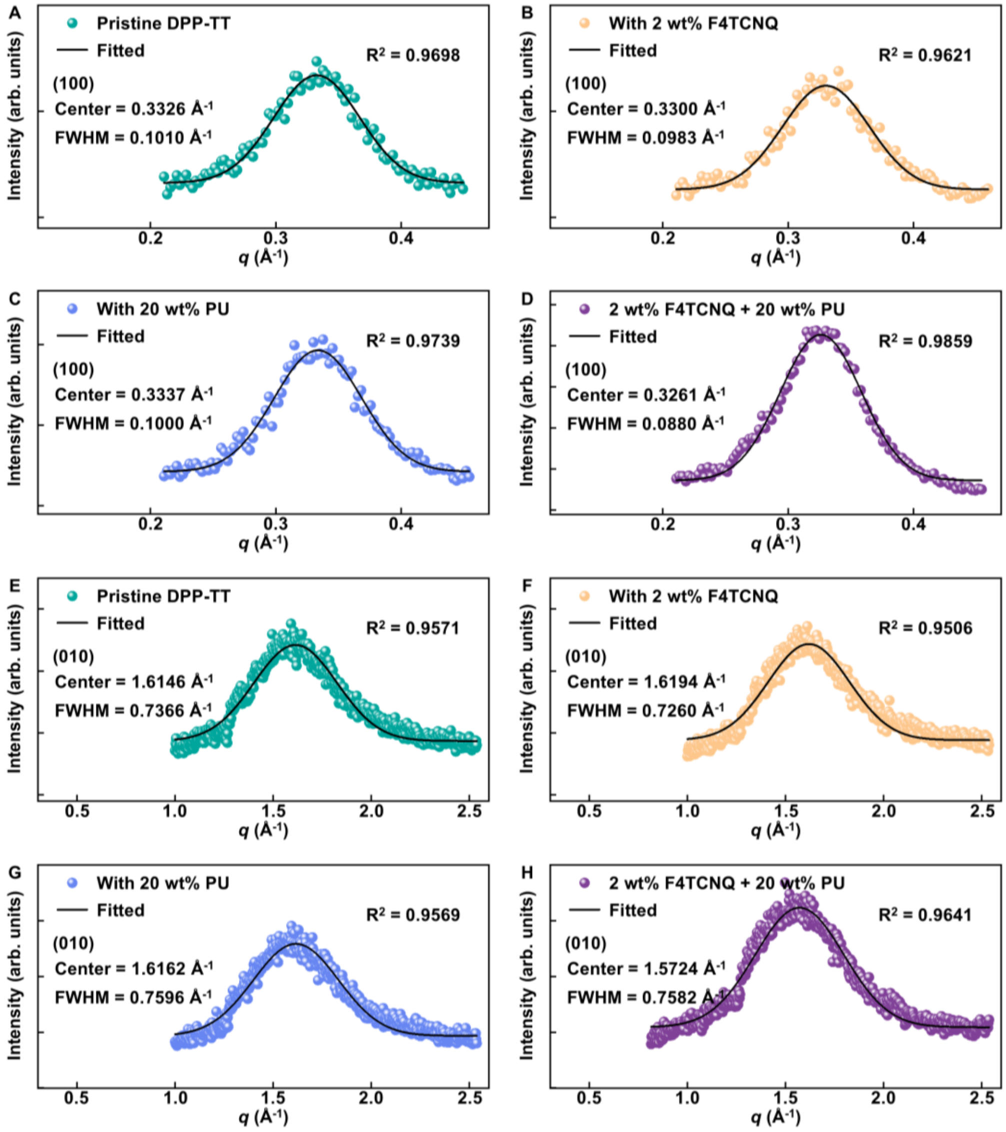

Fig. S24. Lamellar packing and π-π packing of DPP-TT films. (**A** to **D**) Lamellar packing diffraction peak (100) analysis of spin-coated pristine DPP-TT nanofilm (**A**), spin-coated DPP-TT nanofilm doped with 2 wt% F4TCNQ (**B**), rubbery DPP-TT nanofilm with 20 wt% PU (**C**), and rubbery doping DPP-TT nanofilm with 20 wt% PU, 2 wt% F4TCNQ (**D**). (**E** to **H**) π-π packing diffraction peak (010) analysis of spin-coated pristine DPP-TT nanofilm (**E**), spin-coated DPP-TT nanofilm doped with 2 wt% F4TCNQ (**F**), rubbery DPP-TT nanofilm with 20 wt% PU (**G**), and rubbery doping DPP-TT nanofilm with 20 wt% PU, 2 wt% F4TCNQ (**H**).

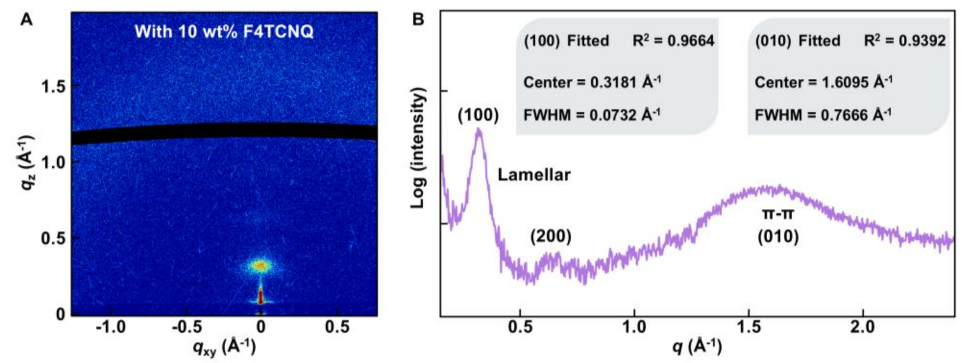

Fig. S25. GIWAXS analysis of spin-coated DPP-TT nanofilm doped with 10 wt% F4TCNQ. 2D GIWAXS pattern (**A**) and the corresponding 1D GIWAXS profile (**B**) of spin-coated DPP-TT nanofilm doped with 10 wt% F4TCNQ.

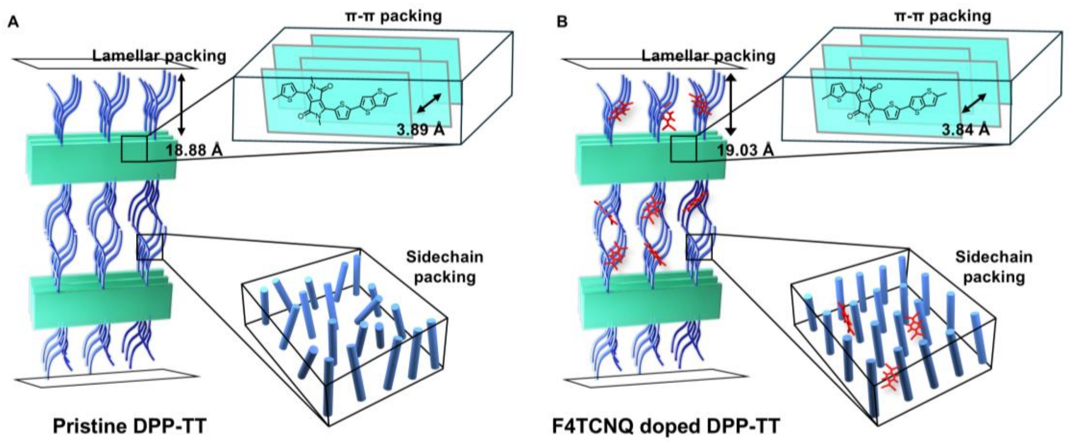

Fig. S26. Solid-state microstructures. Schematic representation of the proposed non-destructive doping concept in the pristine (**A**) and 2 wt% F4TCNQ–doped (**B**) DPP-TT, where green is for DPP-TT backbone, blue for DPP-TT alkyl side chain, and red for dopant.

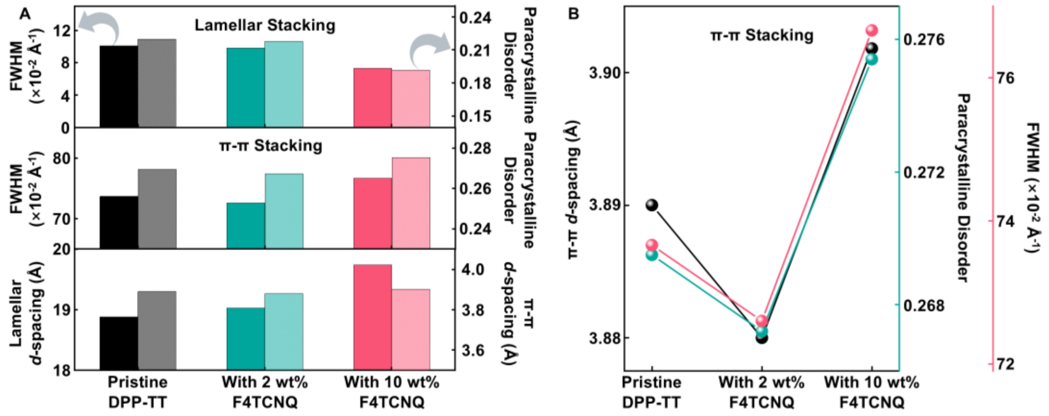

Fig. S27. Lamellar packing and π-π stacking of the pristine DPP-TT and blended nanofilms. (**A**) The analysis for the summarized FWHM, paracrystalline disorder and packing distance on the pristine DPP-TT and blended nanofilms. (**B**) π-π stacking diffraction peak analysis of the pristine DPP-TT and blended nanofilms.

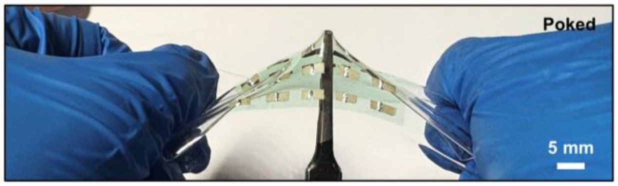

Fig. S28. Rubbery transistor array. Transistor array subjected to a poking deformation mode.

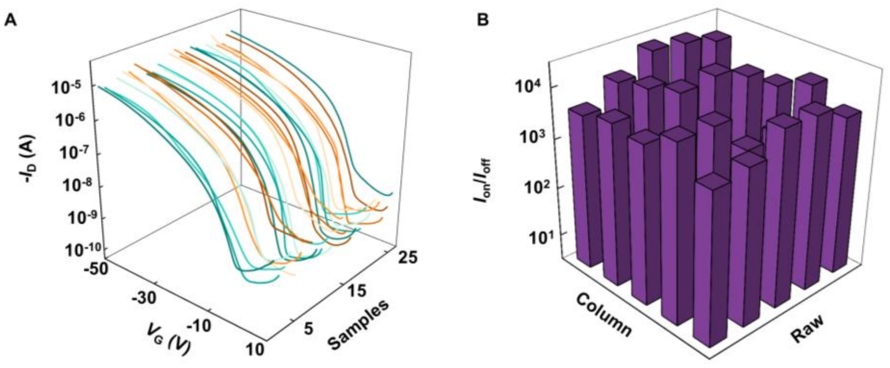

Fig. S29. Electronic performance of fully stretchable transistor array. Transfer curves (**A**) of 25 devices in array with corresponding current on/off ratio (**B**).

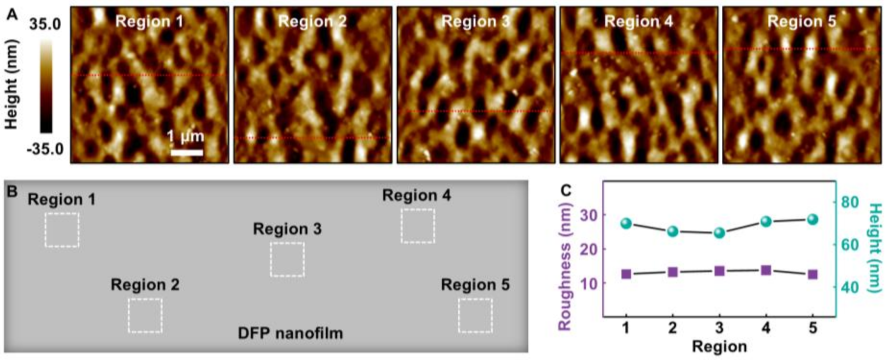

Fig. S30. AFM height images of the DFP nanofilm with 20 wt% PU and 2 wt% F4TCNQ. (**A**, **B**) AFM height images of the five different regions (**A**), corresponding to the schematic shown in (**B**). (**C**) The summary of surface roughness and height.

AFM measurements were taken from five different locations on the constructed DFP nanofilm, all of which show consistent and homogeneous nanoweb architectures (Fig. S30A, B). The obtained roughness and height exhibit acceptable fluctuations across different areas, directly confirming the uniform morphological configuration throughout the entire rubbery semiconductor nanofilm (Fig. S30C).

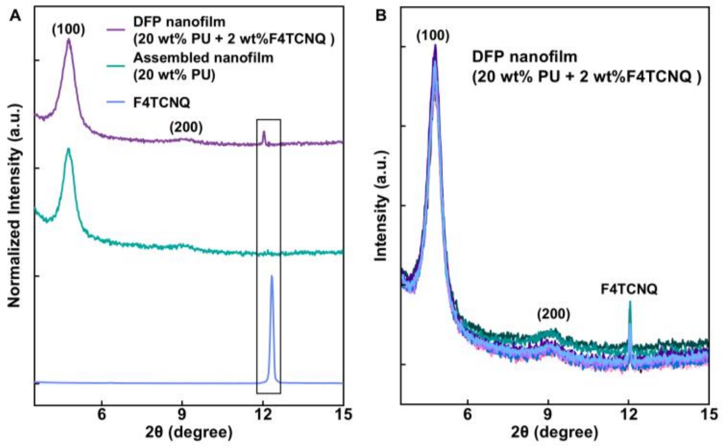

Fig. S31. XRD spectra. (**A**) XRD pattern of the F4TCNQ, rubbery DPP-TT nanofilm with 20 wt% PU and DFP nanofilm with 20 wt% PU, 2 wt% F4TCNQ. (**B**) XRD pattern of the nine different regions in DFP nanofilm.

XRD spectroscopy was used to gain insight into the molecular packing within the nanofilm. The emerging diffraction peak at around 12° revels the successful chemical doping induced by F4TCNQ molecule (Fig. S31A). All nine randomly selected regions show consistent XRD patterns, featuring the characteristic (100) and (200) peaks of DPP-TT and an additional peak arising from F4TCNQ (Fig. S31B). The above results confirm the presence of a highly ordered packing structure within the rubbery semiconducting nanofilms, along with uniform and effective F4TCNQ doping.

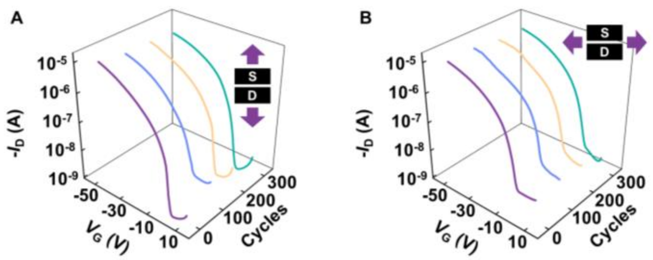

Fig. S32. Rubbery transistors under mechanical strain. (**A**) Representative transfer curves of the rubbery transistor after stretch–release cycles at 30% strain along the channel length direction. (**B**) Representative transfer curves of the rubbery transistor after stretch–release cycles at 30% strain perpendicular to the channel length direction.

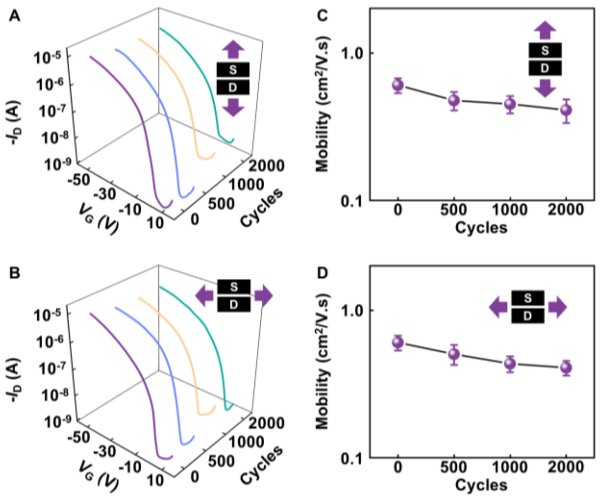

Fig. S33. Rubbery transistors under mechanical strain. (**A**) Representative transfer curves of the rubbery transistor after stretch–release cycles at 30% strain along the channel length direction. (**B**) Representative transfer curves of the rubbery transistor after stretch–release cycles at 30% strain in perpendicular to the channel length. (**C**) Changes in mobility after stretch–release cycles at 30% strain along the channel length. (**D**) Changes in mobility after stretch–release cycles at 30% strain in perpendicular direction to the channel length.

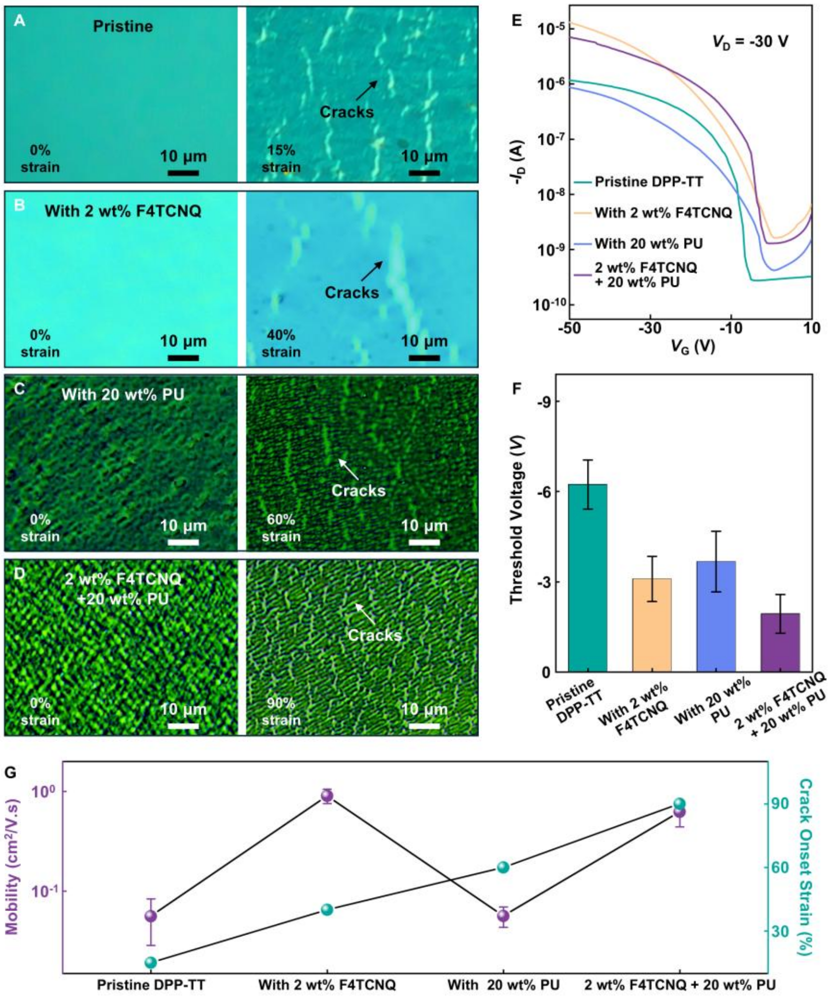

Fig. S34. The analysis for the independent contributions of the PU nanoconfinement and F4TCNQ redox doping on the highly doped rubbery semiconducting nanofilms. (**A-D**) The optical images at 0% and crack onset strain of the pristine DPP-TT and blended nanofilms. (**A**) The optical images of the pristine DPP-TT nanofilms stretched at 0% and 15% strain, respectively. (**B**) The optical images of the blended DPP-TT nanofilms with 2 wt% F4TCNQ stretched at 0% and 40% strain, respectively. (**C**) The optical images of the blended DPP-TT nanofilms with 20 wt% PU stretched at 0% and 60% strain, respectively. (**D**) The optical images of the blended DPP-TT nanofilms with 2 wt% F4TCNQ and 20 wt% PU stretched at 0% and 90% strain, respectively. (**E**) Representative transfer curves of the thin film transistors based on pristine DPP-TT and blended nanofilms. (**F**) Calculated threshold voltage of the thin film transistors based on pristine DPP-TT and blended nanofilms. (**G**) The corresponding mobility and crack onset strain of the pristine DPP-TT and blended nanofilms.

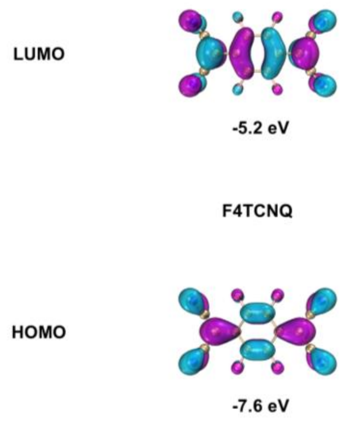

Fig. S35. Calculated energy levels and molecular orbital diagrams of F4TCNQ.

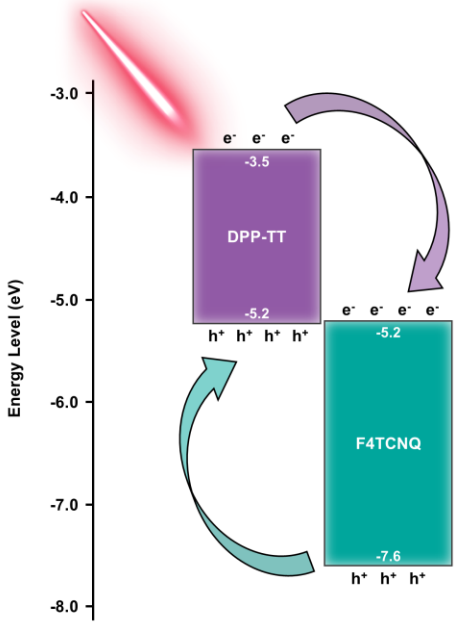

Fig. S36. Energy-level diagram of the heterojunction between DPP-TT and F4TCNQ, and the corresponding photogenerated carrier transfer processes under NIR illumination (32, 47).

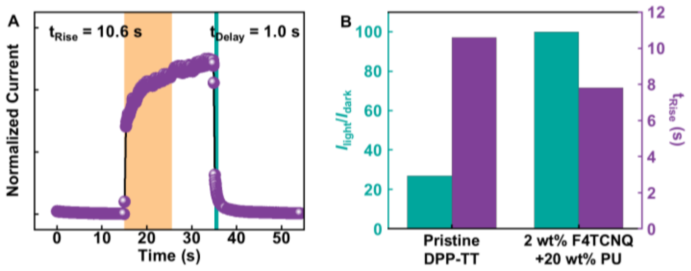

Fig. S37. The analysis for NIR temporal response between pristine DPP-TT nanofilm and highly doped rubbery semiconducting nanofilm with 2 wt% F4TCNQ and 20 wt% PU. (A) Rise and decay times of the pristine DPP-TT nanofilm under the illumination of 5.08 mW/cm^2. (B) The corresponding current on/off ratios and rise times of the pristine DPP-TT and highly doped rubbery semiconducting nanofilm with 2 wt% F4TCNQ and 20 wt% PU.

The slow response actually is a common challenge for stretchable phototransistors (Table S5). The slow response regarding both nanofilms can be explained by underlying physics. Upon illumination, the photocurrent growth exhibits a rapid initial increase followed by a slow rise persisting for several seconds. Specifically, the rise in hole population within the channel during initial illumination lowers the effective barrier height and concentrates on charge tunneling/transport from the DPP-TT layer to the electrodes, substantially leading to the enhanced photocurrent. However, this increase in hole density gives rise to accelerated electron-hole recombination at the interface and the generation of an inverse built-in electric field to equilibrate Fermi levels. These two effects collectively degrade hole injection efficiency, thereby impeding photocurrent growth (41). The observed slow recovery of the photocurrent is primarily due to the slow detrapping or recombination of photogenerated electron-hole pairs after turning off the NIR light (15).

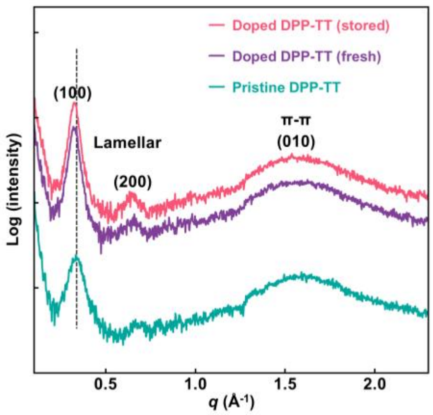

Fig. S38. GIWAXS profiles. GIWAXS profiles of the pristine DPP-TT nanofilm compared with those of the fresh and one month-stored DFP nanofilms.

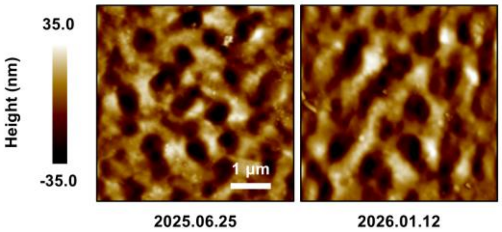

Fig. S39. Long-term stability of the highly doped rubbery semiconducting nanofilm, shown by the comparison of a fresh film with one stored for 6 months. AFM height images of the same DFP nanofilm recorded at different time. The image captured on June 25, 2025 is intentionally reused from Fig. 2I in the main text to maintain logical flow and facilitate direct comparison.

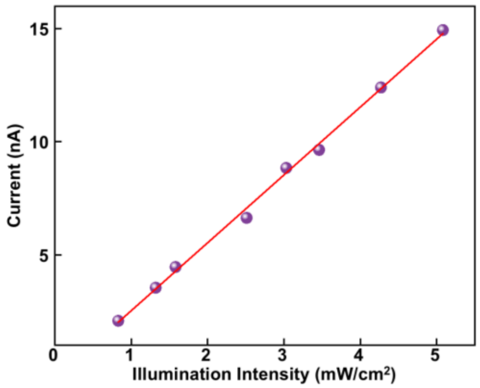

Fig. S40. NIR induced I_{light} versus illumination intensities from 0.83 to 5.08 mW/cm^2 at V_D = -30V (V_G = 0 V).

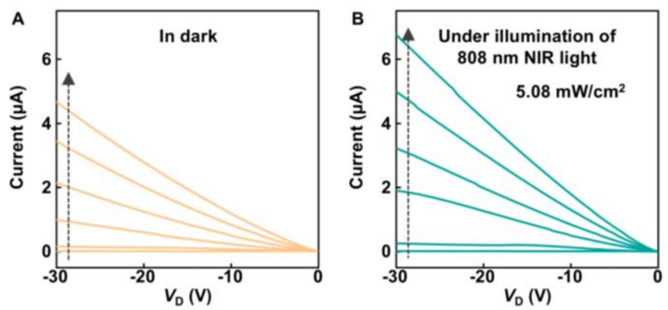

Fig. S41. Photoresponse of rubbery DFP phototransistor. Typical output curves of the rubbery DFP phototransistor conducted in the dark (**A**) and under the illumination of 5.08 mW/cm^2 (**B**) at V_D = -30V.

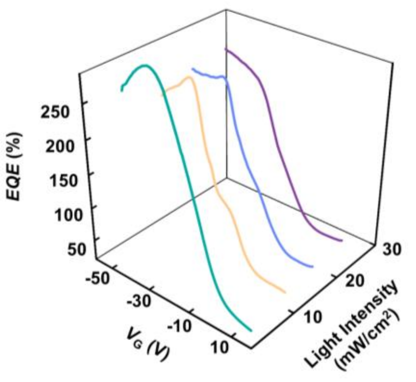

Fig. S42. ***EQE*** **as a function of** ***V*****G for the rubbery DFP phototransistor under various incident illumination intensities.**

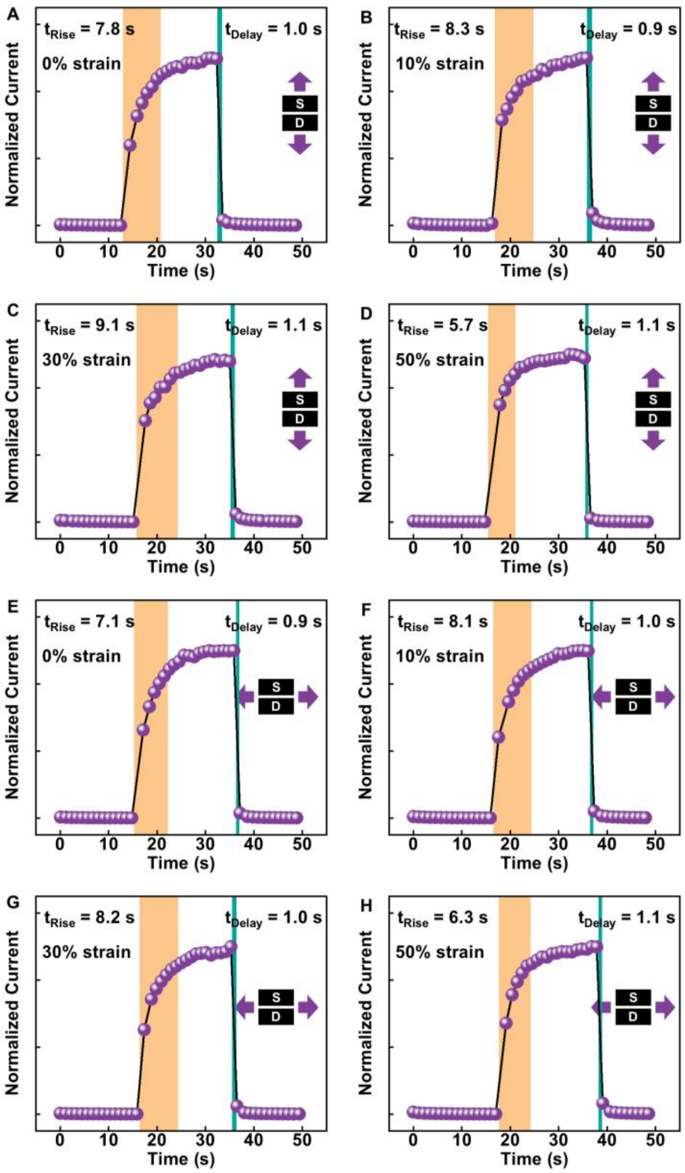

Fig. S43. High-resolution temporal response. (**A** to **H**) Rise and decay times of the rubbery DFP nanofilm under the illumination of 5.08 mW/cm^2 stretched at mechanical strains of 0%, 10%, 30% and 50% along (**A** to **D**) or perpendicular (**E** to **H**) to the channel length directions.

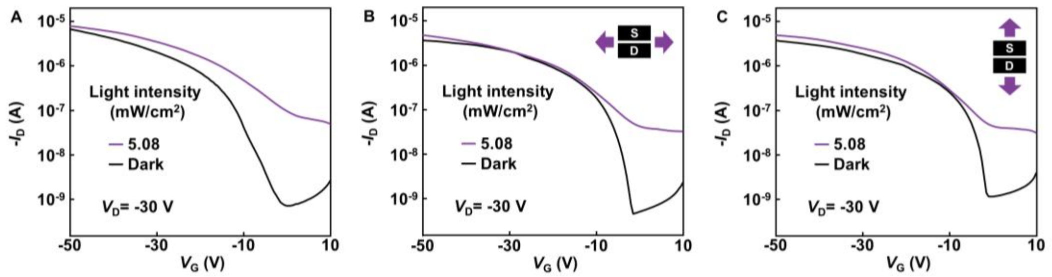

Fig. S44. NIR response of the rubbery DFP nanofilm under multiple stretch–release cycles. (**A**) Typical transfer characteristics of the unstretched rubbery DFP phototransistor conducted in the dark and under the illumination of 5.08 mW/cm^2. (**B**, **C**) Typical transfer characteristics of the rubbery DFP phototransistor conducted in the dark and under the illumination of 5.08 mW/cm^2 after 2000 stretch–release cycles at 30% strain perpendicular (**B**) or parallel (**C**) to the channel length directions.

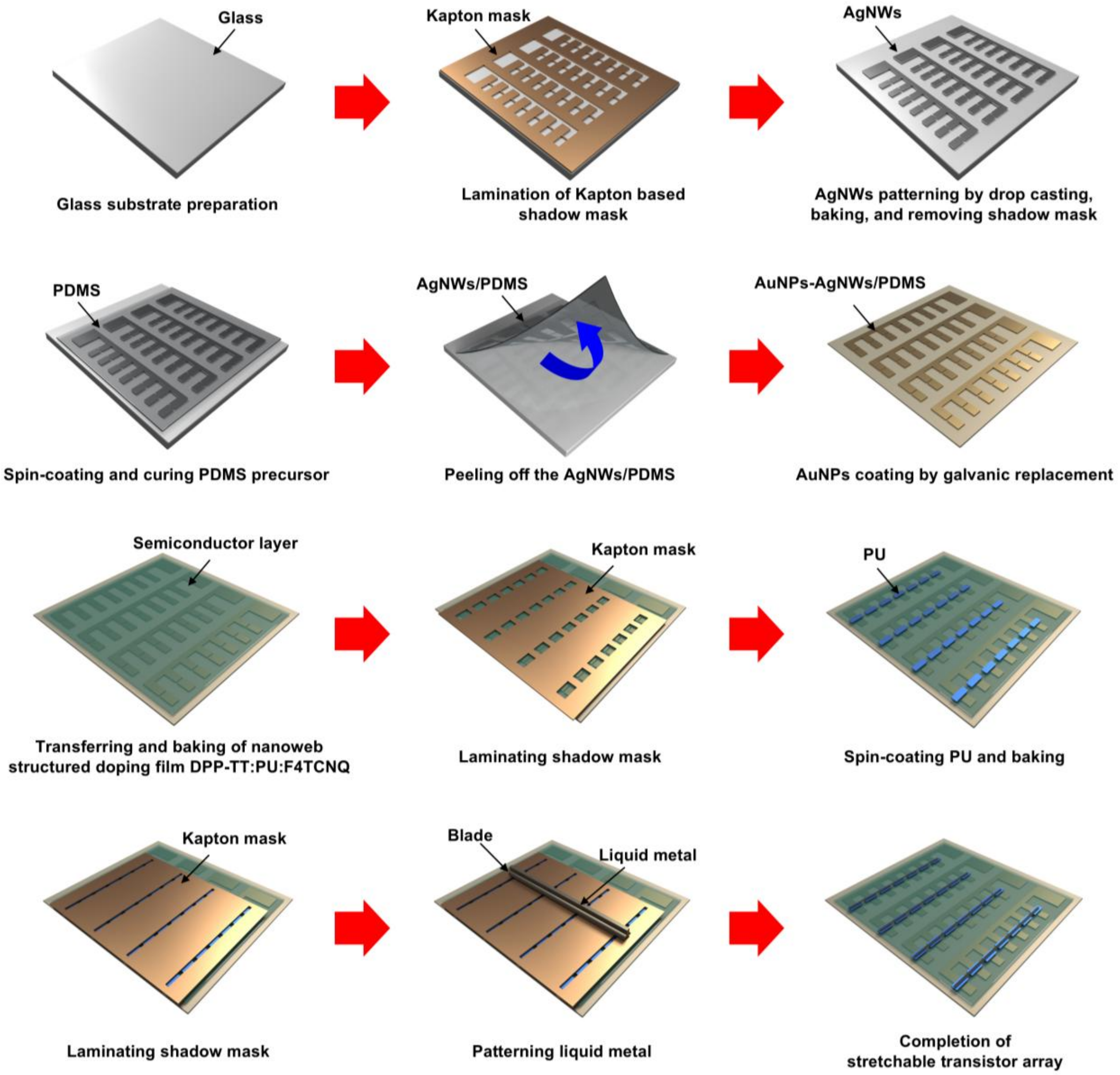

Fig. S45. Fabrication process of the rubbery phototransistor array based on the DFP nanofilm for the NIR imaging.

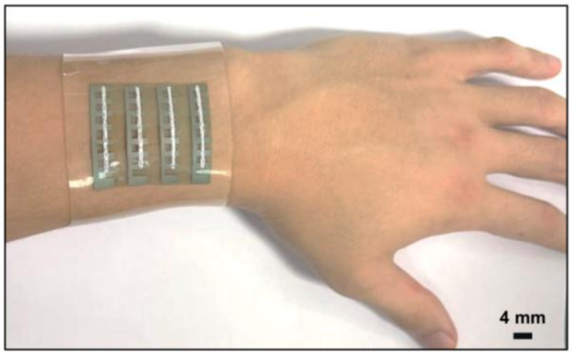

Fig. S46. The rubbery phototransistor array adheres and conforms to the human wrist.

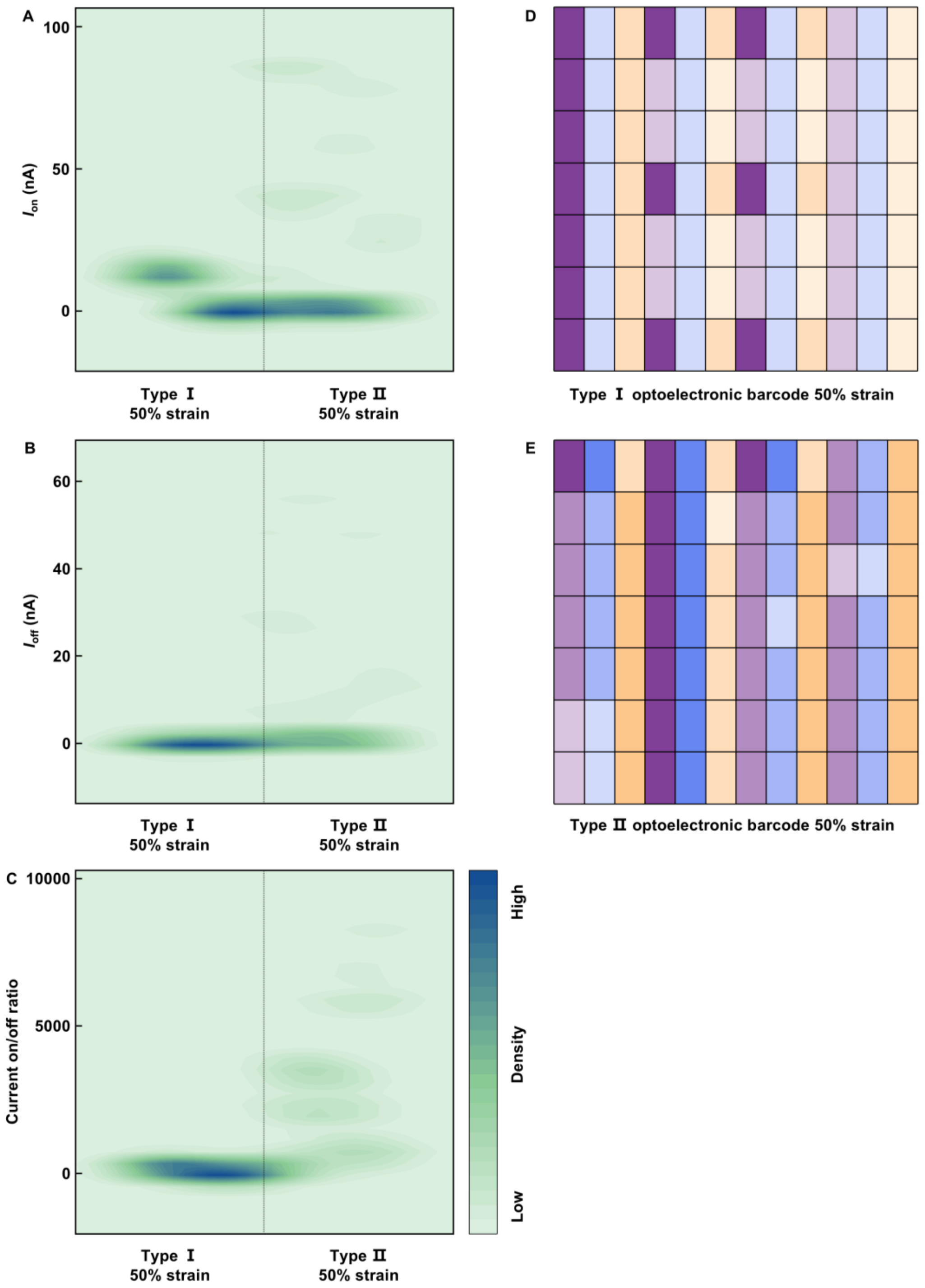

Fig. S47. Stretchable 2D optoelectronic barcodes. (**A** to **C**) The summarized I_{on} (**A**), I_{off} (**B**) and current on/off ratio (**C**) of all pixels in type I and II arrays stretched at 50% strain. Specially, the I_{on} and current on/off of pixels located in the obstructed area in type I array, is designated as I_{off} and 1, respectively. The comprehensive stretchable 2D optoelectronic barcodes operation on rubbery phototransistor type I array (**D**) and type II array (**E**) stretched at 50% strain.

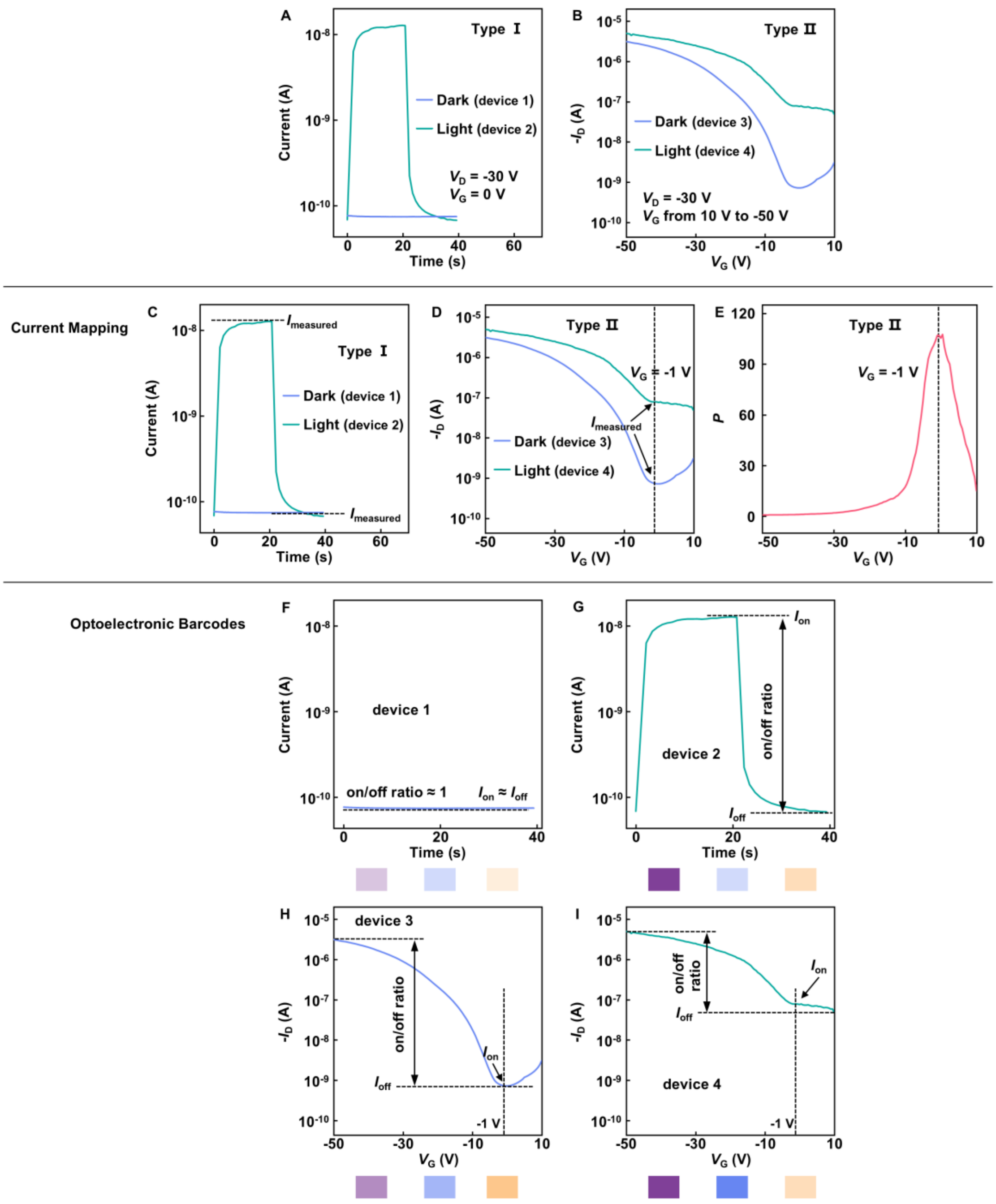

Fig. S48. The specific output indicators and operating conditions of the fabricated NIR imaging system and optoelectronic barcode. (**A**) The recorded temporal responses of two pixels with/without the NIR illumination of 5.08 mW/cm^2 in type I array. (**B**) The recorded transfer curves of two pixels with/without the NIR illumination of 5.08 mW/cm^2 in type II array. (**C**, **D**) The specific $I_{measured}$ in type I mode (**C**) and type II mode (**D**) for current mapping. (**E**) The corresponding P of device 4. (**F**-**I**) The specific I_{on}, I_{off} and current on/off ratio in four devices for optoelectronic barcode. Device 1 (**F**) and device 2 (**G**) belong to type I mode, device 3 (**H**) and device 4 (**I**) belong to type II mode.

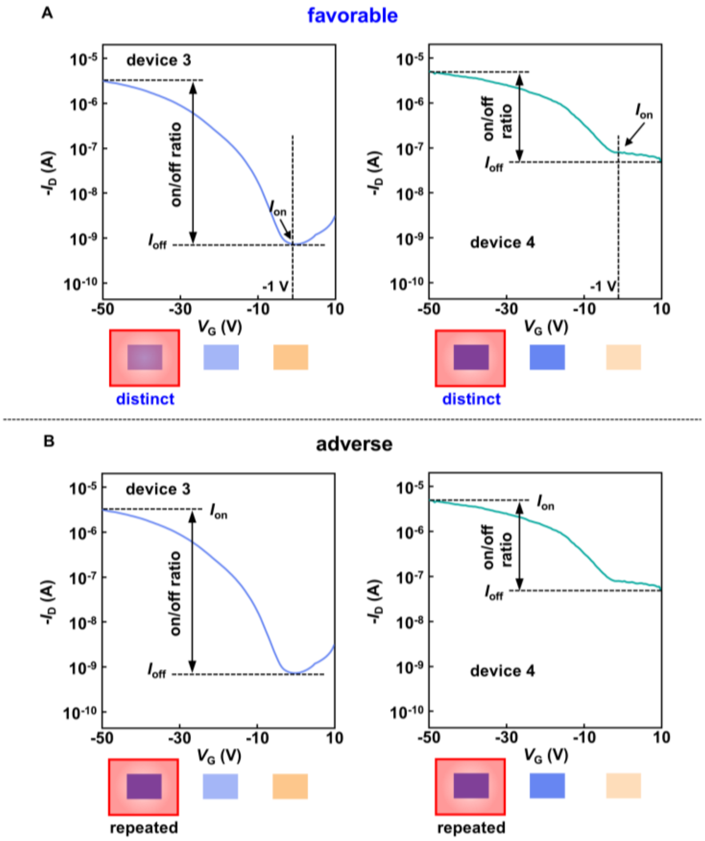

Fig. S49. Illustrations of signal recognition and information contrast. (**A**) The I_{on} extracted from the V_G of -1 V of device 3 and device 4 in type II mode for optoelectronic barcode. (**B**) The I_{on} refers to the largest photocurrent of device 3 and device 4 in type II mode for optoelectronic barcode.

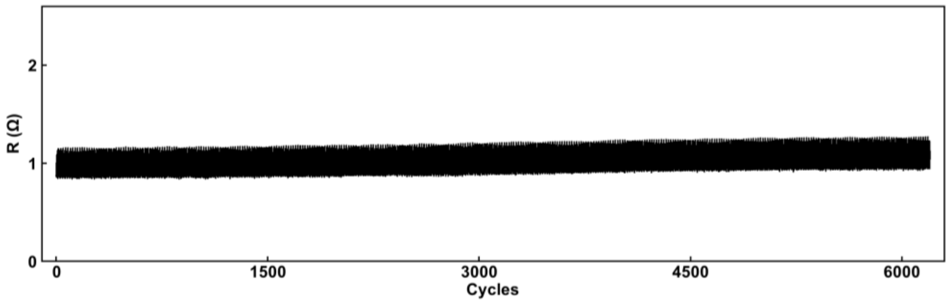

Fig. S50. The high electrical conductivity and excellent cyclic stretching stability of gate electrode in our work. The resistance changes of the liquid metal during more than 6000 cycles of stretching at 50% strain.

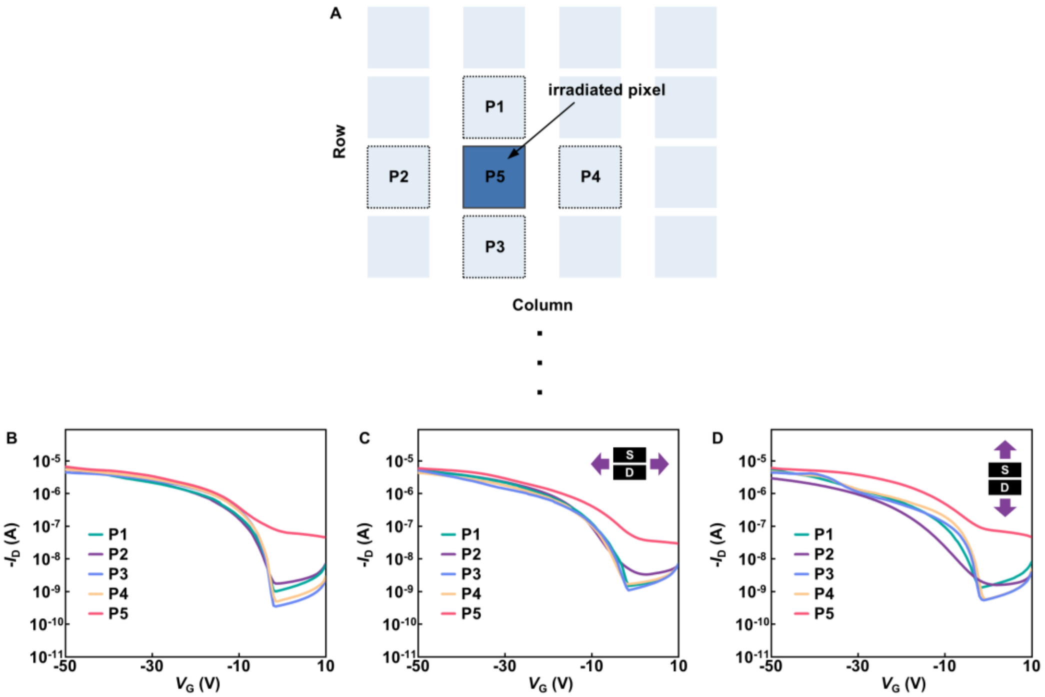

Fig. S51. Evaluation of reliability and crosstalk immunity. (**A**) Signal cross-talk characterization in the matrix. (**B**) Typical transfer curves before stretching. (**C**, **D**) Typical transfer curves under stretching at mechanical strains of 50% perpendicular (**C**) or parallel (**D**) to the channel length directions.

Table S1. The comparative analysis of our air/water interfacial self-assembled rubbery semiconductors with other semiconducting film processing techniques for stretchable transistors.

Semiconducting materials	Technology	Implementation	Initial performance ($cm^2V^{-1}s^{-1}$)	Stretchability	Area	Ref.
DPP-TT/PU/F4TCNQ	air/water interfacial self-assembly	none	0.60	0.41 $cm^2V^{-1}s^{-1}$ (30% strain, 2000 cycles)	25×10 cm^2	this work
DPP-TT/SEBS	spin-coating	spin coater	0.59	0.50 $cm^2V^{-1}s^{-1}$ (25% strain, 1000 cycles)	N/A	Xu et al., 2017 (24)
DPPTT/branched diazirine	spin-coating	spin coater	0.255	50% strain 1000 cycles	N/A	Zheng et al., 2021 (48)
P3HT/SEBS	spin-coating	spin coater	0.005	0.001 $cm^2V^{-1}s^{-1}$ (50% strain, 200 cycles)	N/A	Shin et al., 2015 (49)
DPP-TT/SEBS	spin-coating	spin coater	0.60	25% strain 1000 cycles	N/A	Liu et al., 2023 (50)
P3HT	spin-coating	spin coater	0.034	40% strain 100 cycles	N/A	Chortos et al., 2014 (51)
DPP-TT/SEBS	solution shearing	shearing stage with heated base	1.31	0.45 $cm^2V^{-1}s^{-1}$ (35% strain, 1000 cycles)	10×10 cm^2	Li et al., 2024 (52)
DPP-TT/SBS	solution shearing	shearing stage with heated base	0.27	40% strain 500 cycles	1×3 cm^2	Yan et al., 2020 (53)
DB-TTF/PS	solution shearing	shearing stage with heated base	0.27	flexible	6×6 cm^2	Georgakopoulos et al., 2015 (54)
DPPDTSE/SEBS	roll-to-roll	slot die coater and microtrench shearing blade	1.5	50% strain 1000 cycles	metre-scale	Xu et al., 2019 (26)
Merck SP400	roll-to-roll	gravure printing	0.11	N/A	no mention	Lee et al., 2018 (55)
Triarylamine copolymer	roll-to-roll	screen printing and gravure printing	0.26	N/A	no mention	Verilhac et al., 2010 (56)
P3HT	roll-to-roll	planar gravure cliche	0.04	N/A	no mention	Voigt et al., 2010 (57)
ink SP series from Merck	Plate-to-roll	plate-to-roll gravure printing	0.21	N/A	no mention	Kim et al., 2017 (58)
diF-TES-ADT	solution bar-coating	wire-wound bar	0.2	N/A	4-inch wafer	Sung et al., 2020 (59)
pentacene	electrohydro dynamic printing	home-built automatic printing system	0.52	flexible	4-inch wafer	Kim et al., 2020 (60)

Table S2. A systematic comparison of the electronic and mechanical behaviors about different nanofilms fabricated in this work.

Nanofilm	Mobility ($cm^2 V^{-1} s^{-1}$)	Threshold voltage (V)	Crack onset strain (%)
Pristine DPP-TT	~ 0.0561	~ -6.23	15
With 2 wt% F4TCNQ	~ 0.904	~ -3.09	40
With 20 wt% PU	~ 0.0564	~ -3.67	60
2 wt% F4TCNQ+20 wt% PU	~ 0.625	~ -1.93	90

Table S3. The comparative analysis of our rubbery phototransistors with other state-of-the-art deformable phototransistors in the literature, in terms of semiconducting materials, optoelectronic performance and figures of merit.

Semiconductors	Detection wavelength (nm)	Responsivity (A/W)	Detectivity (Jones)	Stretchability	t_{Rise}/t_{Delay} (ms)	Operation mode	Ref.
DPP-TT/PU/F4TCNQ	NIR (808 nm)	2	10^{11}	30% strain 2000 cycles	7800 1000	multiplexable	this work
CdSe/DPP-TT/SEBS	UV to NIR	5.74×10^{-5} (450 nm)	1.392×10^{7} (450 nm)	30% strain 1000 cycles	310 270	multiplexable	Song et al., 2022 (14)
$CsPbBr_3$/DPP-TT/SEBS	X-ray to UV	2600 (365 nm)	5.5×10^{14} (365 nm)	25% strain 1000 cycles	3900 19900	single cell	Liu et al., 2022 (41)
IDT-BT/SEBS	white light	no mention	no mention	100% strain (not cyclic)	no mention	single cell	Liu et al., 2021 (61)
PQT-12:PEO/PU:PEO	Vis	58.2 (470 nm)	no mention	30% strain (not cyclic)	no mention	single cell	Lee et al., 2021 (62)
PSC/QDs/PSC	Vis to NIR	1.62×10^{-5} (830 nm)	no mention	50% strain 10000 cycles	300 400	single cell	Nam et al., 2024 (63)
DPP-DTT	Vis to NIR	0.18 (808 nm)	3.94×10^{6} (808 nm)	30% strain (not cyclic)	11100 9630	single cell	Guo et al., 2023 (64)
DPP-TT/PVA/DPP-TT	NIR (808 nm)	33.3	2.22×10^{13}	N/A	no mention	multiplexable	Yu et al., 2026 (65)
PTCDI-C13	Vis	28 (489 nm)	no mention	N/A	no mention	single cell	Liu et al., 2018 (66)
MoS_2	Vis	0.02 (405 nm)	4.8×10^{7} (405 nm)	N/A	no mention	single cell	Kim et al., 2017 (67)
PolyTPD:BCF/P3HT	NIR	0.538 (1500 nm)	no mention	N/A	no mention	single cell	Lee et al., 2021 (68)
PQT-12:PEO	Vis	0.93 (470 nm)	no mention	N/A	no mention	multiplexable	Lee et al., 2016 (69)
PEHTPPD-BT:P3HT	Vis to NIR	0.25 (900 nm)	no mention	N/A	no mention	single cell	Han et al., 2015 (70)
PDVT-8:PCBM	Vis (720 nm)	750	4.5×10^{15}	N/A	720 30	single cell	Zhong et al., 2018 (71)
C8-BTBT:PLA	UV (365 nm)	393	no mention	N/A	no mention	single cell	Huang et al., 2017 (72)
rGO-PEDOT:PSS/WS_2 QDs	UV to Vis	4.42×10^{2} (365 nm)	2.86×10^{11} (365 nm)	N/A	1780 2910	single cell	Hao et al., 2024 (73)

Table S4. Comparison of this work with different encoding methods.

Classification	Principle	Advantage/Limitation	Stretchability	Ref.
Multiplexed phototransistor array	information coding utilizing the electrical response induced by NIR radiation and bias voltage sensitivity	high reading accuracy, high encoding capacity, high security level, excellent optoelectronic sensitivity, and mechanical compatibility with curved surfaces	Stretchable	this work
Programmable flow lithography technique	precisely controlled microarchitectures of photopatterning as codes	abundant coding capacity/<br>high cost of the equipment, complex operation procedures, slow speed, and processing volume	no mention	Lee et al., 2010 (74);<br>Lee et al., 2014 (75);<br>Yoo et al., 2025 (76)
Epitaxial self-assembled microcrystals	different emissions in a single microstructure as logical codes	high reading accuracy, organization designability/<br>limited encoding ability	rigid	Sun et al., 2018 (77);<br>Lv et al., 2024 (78)
Template assisted electrodeposition	electrodeposition metal ions into templates as codes	abundant design ability/<br>limited encoding ability, complex operation procedures, and misinterpretation risk	no mention	Nicewarner-Peña et al., 2001 (79);<br>Keating et al., 2003 (80);<br>Widerker et al., 2024 (81)
Inkjet printing	pre-designed patterns were fabricated as readable codes via direct printing	abundant design ability, covert communication/<br>decoding is rather complex and misinterpretation risk	flexible	Liu et al., 2019 (82);<br>Zub et al., 2022 (83)
Spatial selective photobleaching	encoding digital information directly onto individual micro-nano materials using photobleaching patterns	unlimited number of unique codes/<br>the bleached code decays or fades with time	no mention	Braeckmans et al., 2003 (84);<br>Huang et al., 2010 (85);<br>Catalano et al., 2025 (86)

REFERENCES

1. X. Du, J. Han, Z. He, C. Han, X. Wang, J. Wang, Y. Jiang, S. Tao, Efficient organic upconversion devices for low energy consumption and high-quality noninvasive imaging. *Adv. Mater.* **33**, e2102812 (2021).

2. X. Gao, Y. Cui, R. M. Levenson, L. W. K. Chung, S. Nie, In vivo cancer targeting and imaging with semiconductor quantum dots. *Nat. Biotechnol.* **22**, 969–976 (2004).

3. H. Yu, D. Kim, J. Lee, S. Baek, J. Lee, R. Singh, F. So, High-gain infrared-to-visible upconversion light-emitting phototransistors. *Nat. Photonics* **10**, 129–134 (2016).

4. P. C. Y. Chow, T. Someya, Organic photodetectors for next-generation wearable electronics. *Adv. Mater.* **32**, e1902045 (2020).

5. X. Wang, Z. Cheng, K. Xu, H. K. Tsang, J.-B. Xu, High-responsivity graphene/silicon-heterostructure waveguide photodetectors. *Nat. Photonics* **7**, 888–891 (2013).

6. T. Yokota, T. Nakamura, H. Kato, M. Mochizuki, M. Tada, M. Uchida, S. Lee, M. Koizumi, W. Yukita, A. Takimoto, T. Someya, A conformable imager for biometric authentication and vital sign measurement. *Nat. Electron.* **3**, 113–121 (2020).

7. W. Lee, Y. Liu, Y. Lee, B. K. Sharma, S. M. Shinde, S. D. Kim, K. Nan, Z. Yan, M. Han, Y. Huang, Y. Zhang, J.-H. Ahn, J. A. Rogers, Two-dimensional materials in functional three-dimensional architectures with applications in photodetection and imaging. *Nat. Commun.* **9**, 1417 (2018).

8. H. Shim, K. Sim, B. Wang, Y. Zhang, S. Patel, S. Jang, T. J. Marks, A. Facchetti, C. Yu, Elastic integrated electronics based on a stretchable n-type elastomer–semiconductor–elastomer stack. *Nat. Electron.* **6**, 349–359 (2023).

9. J. Hu, W.-C. Gao, Y.-D. Zhao, B. Fan, X. Sun, J. Qiao, Y.-S. Guan, Q. Li, Broadband-responsive rubbery stretchable vertical-structured photodetectors based on rubbery stretchable transparent conductors. *ACS Nano* **19**, 18347–18356 (2025).

10. C. Wang, X. Ren, C. Xu, B. Fu, R. Wang, X. Zhang, R. Li, H. Li, H. Dong, Y. Zhen, S. Lei, L. Jiang, W. Hu, N-type 2D organic single crystals for high-performance organic field-effect transistors and near-infrared phototransistors. *Adv. Mater.* **30**, e1706260 (2018).

11. L. Gu, S. Poddar, Y. Lin, Z. Long, D. Zhang, Q. Zhang, L. Shu, X. Qiu, M. Kam, A. Javey, Z. Fan, A biomimetic eye with a hemispherical perovskite nanowire array retina. *Nature* **581**, 278–282 (2020).

12. M. Kim, G. J. Lee, C. Choi, M. S. Kim, M. Lee, S. Liu, K. W. Cho, H. M. Kim, H. Cho, M. K. Choi, N. Lu, Y. M. Song, D.-H. Kim, An aquatic-vision-inspired camera based on a monocentric lens and a silicon nanorod photodiode array. *Nat. Electron.* **3**, 546–553 (2020).

13. Z. Rao, Y. Lu, Z. Li, K. Sim, Z. Ma, J. Xiao, C. Yu, Curvy, shape-adaptive imagers based on printed optoelectronic pixels with a kirigami design. *Nat. Electron.* **4**, 513–521 (2021).

14. J.-K. Song, J. Kim, J. Yoon, J. H. Koo, H. Jung, K. Kang, S.-H. Sunwoo, S. Yoo, H. Chang, J. Jo, W. Baek, S. Lee, M. Lee, H. J. Kim, M. Shin, Y. J. Yoo, Y. M. Song, T. Hyeon, D.-H. Kim, D. Son, Stretchable colour-sensitive quantum dot nanocomposites for shape-tunable multiplexed phototransistor arrays. *Nat. Nanotechnol.* **17**, 849–856 (2022).

15. Y. Bian, K. Liu, Y. Ran, Y. Li, Y. Gao, Z. Zhao, M. Shao, Y. Liu, J. Kuang, Z. Zhu, M. Qin, Z. Pan, M. Zhu, C. Wang, H. Chen, J. Li, X. Li, Y. Liu, Y. Guo, Spatially nanoconfined N-type polymer semiconductors for stretchable ultrasensitive X-ray detection. *Nat. Commun.* **13**, 7163 (2022).

16. Y. Bian, M. Zhu, C. Wang, K. Liu, W. Shi, Z. Zhu, M. Qin, F. Zhang, Z. Zhao, H. Wang, Y. Liu, Y. Guo, A detachable interface for stable low-voltage stretchable transistor arrays and high-resolution X-ray imaging. *Nat. Commun.* **15**, 2624 (2024).

17. Y.-S. Guan, F. Ershad, Z. Rao, Z. Ke, E. C. da Costa, Q. Xiang, Y. Lu, X. Wang, J. Mei, P. Vanderslice, C. Hochman-Mendez, C. Yu, Elastic electronics based on micromesh-structured rubbery semiconductor films. *Nat. Electron.* **5**, 881–892 (2022).

18. R. Noriega, J. Rivnay, K. Vandewal, F. P. V. Koch, N. Stingelin, P. Smith, M. F. Toney, A. Salleo, A general relationship between disorder, aggregation and charge transport in conjugated polymers. *Nat. Mater.* **12**, 1038–1044 (2013).

19. Y.-Y. Zhou, Y.-C. Xu, Z.-F. Yao, J.-Y. Li, C.-K. Pan, Y. Lu, C.-Y. Yang, L. Ding, B.-F. Xiao, X.-Y. Wang, Y. Shao, W.-B. Zhang, J.-Y. Wang, H. Wang, J. Pei, Visualizing the multi-level assembly structures of conjugated molecular systems with chain-length dependent behavior. *Nat. Commun.* **14**, 3340 (2023).

20. W. Wang, Y. Jiang, D. Zhong, Z. Zhang, S. Choudhury, J.-C. Lai, H. Gong, S. Niu, X. Yan, Y. Zheng, C.-C. Shih, R. Ning, Q. Lin, D. Li, Y.-H. Kim, J. Kim, Y.-X. Wang, C. Zhao, C. Xu, X. Ji, Y. Nishio, H. Lyu, J. B.-H. Tok, Z. Bao, Neuromorphic sensorimotor loop embodied by monolithically integrated, low-voltage, soft e-skin. *Science* **380**, 735–742 (2023).

21. K. Sim, Z. Rao, H.-J. Kim, A. Thukral, H. Shim, C. Yu, Fully rubbery integrated electronics from high effective mobility intrinsically stretchable semiconductors. *Sci. Adv.* **5**, eaav5749 (2019).

22. J. Xu, H.-C. Wu, J. Mun, R. Ning, W. Wang, G.-J. N. Wang, S. Nikzad, H. Yan, X. Gu, S. Luo, D. Zhou, J. B.-H. Tok, Z. Bao, Tuning conjugated polymer chain packing for stretchable semiconductors. *Adv. Mater.* **34**, e2104747 (2022).

23. H. Wei, Z. Cheng, T. Wu, Y. Liu, J. Guo, P.-A. Chen, J. Xia, H. Xie, X. Qiu, T. Liu, B. Zhang, J. Hui, Z. Zeng, Y. Bai, Y. Hu, Novel organic superbase dopants for ultraefficient N-doping of organic semiconductors. *Adv. Mater.* **35**, e2300084 (2023).

24. J. Xu, S. Wang, G.-J. N. Wang, C. Zhu, S. Luo, L. Jin, X. Gu, S. Chen, V. R. Feig, J. W. F. To, S. Rondeau-Gagné, J. Park, B. C. Schroeder, C. Lu, J. Y. Oh, Y. Wang, Y.-H. Kim, H. Yan, R. Sinclair, D. Zhou, G. Xue, B. Murmann, C. Linder, W. Cai, J. B.-H. Tok, J. W. Chung, Z. Bao, Highly stretchable polymer semiconductor films through the nanoconfinement effect. *Science* **355**, 59–64 (2017).

25. D. Zhong, C. Wu, Y. Jiang, Y. Yuan, M.-g. Kim, Y. Nishio, C.-C. Shih, W. Wang, J.-C. Lai, X. Ji, T. Z. Gao, Y.-X. Wang, C. Xu, Y. Zheng, Z. Yu, H. Gong, N. Matsuhisa, C. Zhao, Y. Lei, D. Liu, S. Zhang, Y. Ochiai, S. Liu, S. Wei, J. B. H. Tok, Z. Bao, High-speed and large-scale intrinsically stretchable integrated circuits. *Nature* **627**, 313–320 (2024).

26. J. Xu, H.-C. Wu, C. Zhu, A. Ehrlich, L. Shaw, M. Nikolka, S. Wang, F. Molina-Lopez, X. Gu, S. Luo, D. Zhou, Y.-H. Kim, G.-J. N. Wang, K. Gu, V. R. Feig, S. Chen, Y. Kim, T. Katsumata, Y.-Q. Zheng, H. Yan, J. W. Chung, J. Lopez, B. Murmann, Z. Bao, Multi-scale ordering in highly stretchable polymer semiconducting films. *Nat. Mater.* **18**, 594–601 (2019).

27. Y. Li, N. Li, W. Liu, A. Prominski, S. Kang, Y. Dai, Y. Liu, H. Hu, S. Wai, S. Dai, Z. Cheng, Q. Su, P. Cheng, C. Wei, L. Jin, J. A. Hubbell, B. Tian, S. Wang, Achieving tissue-level softness on stretchable electronics through a generalizable soft interlayer design. *Nat. Commun.* **14**, 4488 (2023).

28. M. H. Kim, M. W. Jeong, J. S. Kim, T. U. Nam, N. T. P. Vo, L. Jin, T. I. Lee, J. Y. Oh, Mechanically robust stretchable semiconductor metallization for skin-inspired organic transistors. *Sci. Adv.* **8**, eade2988 (2022).

29. Y.-S. Guan, A. Thukral, S. Zhang, K. Sim, X. Wang, Y. Zhang, F. Ershad, Z. Rao, F. Pan, P. Wang, J. Xiao, C. Yu, Air/water interfacial assembled rubbery semiconducting nanofilm for fully rubbery integrated electronics. *Sci. Adv.* **6**, eabb3656 (2020).

30. R. Tadmor, Marangoni flow revisited. *J. Colloid Interface Sci.* **332**, 451–454 (2009).

31. Y. D. Zhao, W. Jiang, S. Zhuo, B. Wu, P. Luo, W. Chen, M. Zheng, J. Hu, K.-Q. Zhang, Z.-S. Wang, L.-S. Liao, M.-P. Zhuo, Stretchable photothermal membrane of NIR-II charge-transfer cocrystal for wearable solar thermoelectric power generation. *Sci. Adv.* **9**, eadh8917 (2023).

32. D. Kiefer, R. Kroon, A. I. Hofmann, H. Sun, X. Liu, A. Giovannitti, D. Stegerer, A. Cano, J. Hynynen, L. Yu, Y. Zhang, D. Nai, T. F. Harrelson, M. Sommer, A. J. Moulé, M. Kemerink, S. R. Marder, I. McCulloch, M. Fahlman, S. Fabiano, C. Müller, Double doping of conjugated polymers with monomer molecular dopants. *Nat. Mater.* **18**, 149–155 (2019).

33. Y.-C. Weng, C.-C. Kang, T.-W. Chang, Y.-T. Tsai, S. Khan, T.-M. Hung, C.-C. Shih, Design principles for enhancing both carrier mobility and stretchability in polymer semiconductors via Lewis acid doping. *Adv. Mater.* **37**, e2411572 (2025).

34. F. Zhang, E. Mohammadi, G. Qu, X. Dai, Y. Diao, Orientation-dependent host–dopant interactions for manipulating charge transport in conjugated polymers. *Adv. Mater.* **32**, e2002823 (2020).

35. G.-J. N. Wang, F. Molina-Lopez, H. Zhang, J. Xu, H.-C. Wu, J. Lopez, L. Shaw, J. Mun, Q. Zhang, S. Wang, A. Ehrlich, Z. Bao, Nonhalogenated solvent processable and printable high-performance polymer semiconductor enabled by isomeric nonconjugated flexible linkers. *Macromolecules* **51**, 4976–4985 (2018).

36. J. Mun, J. Kang, Y. Zheng, S. Luo, H.-C. Wu, N. Matsuhisa, J. Xu, G.-J. N. Wang, Y. Yun, G. Xue, J. B.-H. Tok, Z. Bao, Conjugated carbon cyclic nanorings as additives for intrinsically stretchable semiconducting polymers. *Adv. Mater.* **31**, e1903912 (2019).

37. N. T. P. Vo, T. U. Nam, M. W. Jeong, J. S. Kim, K. H. Jung, Y. Lee, G. Ma, X. Gu, J. B. H. Tok, T. I. Lee, Z. Bao, J. Y. Oh, Autonomous self-healing supramolecular polymer transistors for skin electronics. *Nat. Commun.* **15**, 3433 (2024).

38. C.-Y. Yang, Y.-F. Ding, D. Huang, J. Wang, Z.-F. Yao, C.-X. Huang, Y. Lu, H.-I. Un, F.-D. Zhuang, J.-H. Dou, C.-a. Di, D. Zhu, J.-Y. Wang, T. Lei, J. Pei, A thermally activated and highly miscible dopant for n-type organic thermoelectrics. *Nat. Commun.* **11**, 3292 (2020).

39. R. A. Marcus, Electron transfer reactions in chemistry. Theory and experiment. *Rev. Mod. Phys.* **65**, 599–610 (1993).

40. Y. Yamashita, J. Tsurumi, M. Ohno, R. Fujimoto, S. Kumagai, T. Kurosawa, T. Okamoto, J. Takeya, S. Watanabe, Efficient molecular doping of polymeric semiconductors driven by anion exchange. *Nature* **572**, 634–638 (2019).

41. K. Liu, Y. Bian, J. Kuang, X. Huang, Y. Li, W. Shi, Z. Zhu, G. Liu, M. Qin, Z. Zhao, X. Li, Y. Guo, Y. Liu, Ultrahigh-performance optoelectronic skin based on intrinsically stretchable perovskite-polymer heterojunction transistors. *Adv. Mater.* **34**, e2107304 (2022).

42. H.-J. Kim, K. Sim, A. Thukral, C. Yu, Rubbery electronics and sensors from intrinsically stretchable elastomeric composites of semiconductors and conductors. *Sci. Adv.* **3**, e1701114 (2017).

43. T. Lu, F. Chen, Multiwfn: A multifunctional wavefunction analyzer. *J. Comput. Chem.* **33**, 580–592 (2012).

44. M. Xiao, X. Ren, K. Ji, S. Chung, X. Shi, J. Han, Z. Yao, X. Tao, S. J. Zelewski, M. Nikolka, Y. Zhang, Z. Zhang, Z. Wang, N. Jay, I. Jacobs, W. Wu, H. Yu, Y. Abdul Samad, S. D. Stranks, B. Kang, K. Cho, J. Xie, H. Yan, S. Chen, H. Sirringhaus, Achieving ideal transistor characteristics in conjugated polymer semiconductors. *Sci. Adv.* **9**, eadg8659 (2023).

45. D. Jung, C. Lim, H. J. Shim, Y. Kim, C. Park, J. Jung, S. I. Han, S.-H. Sunwoo, K. W. Cho, G. D. Cha, D. C. Kim, J. H. Koo, J. H. Kim, T. Hyeon, D.-H. Kim, Highly conductive and elastic nanomembrane for skin electronics. *Science* **373**, 1022–1026 (2021).

46. G. M. Whitesides, B. Grzybowski, Self-assembly at all scales. *Science* **295**, 2418–2421 (2002).

47. J. Li, Y. Zhao, H. S. Tan, Y. Guo, C.-A. Di, G. Yu, Y. Liu, M. Lin, S. H. Lim, Y. Zhou, H. Su, B. S. Ong, A stable solution-processed polymer semiconductor with record high-mobility for printed transistors. *Sci. Rep.* **2**, 754 (2012).

48. Y.-Q. Zheng, Y. Liu, D. Zhong, S. Nikzad, S. Liu, Z. Yu, D. Liu, H.-C. Wu, C. Zhu, J. Li, H. Tran, J. B.-H. Tok, Z. Bao, Monolithic optical microlithography of high-density elastic circuits. *Science* **373**, 88–94 (2021).

49. M. Shin, J. Y. Oh, K.-E. Byun, Y.-J. Lee, B. Kim, H.-K. Baik, J.-J. Park, U. Jeong, Polythiophene nanofibril bundles surface-embedded in elastomer: A route to a highly stretchable active channel layer. *Adv. Mater.* **27**, 1255–1261 (2015).

50. K. Liu, C. Wang, B. Liu, Y. Bian, J. Kuang, Y. Hou, Z. Pan, G. Liu, X. Huang, Z. Zhu, M. Qin, Z. Zhao, C. Jiang, Y. Liu, Y. Guo, Low-voltage intrinsically stretchable organic transistor amplifiers for ultrasensitive electrophysiological signal detection. *Adv. Mater.* **35**, e2207006 (2023).

51. A. Chortos, J. Lim, J. W. F. To, M. Vosgueritchian, T. J. Dusseault, T.-H. Kim, S. Hwang, Z. Bao, Highly stretchable transistors using a microcracked organic semiconductor. *Adv. Mater.* **26**, 4253–4259 (2014).

52. X. Li, A. Sabir, X. Zhang, H. Jiang, W. Wang, X. Zheng, H. Yang, Highly stretchable and oriented wafer-scale semiconductor films for organic phototransistor arrays. *ACS Appl. Mater. Interfaces* **16**, 36678–36687 (2024).

53. Q.-Y. Yan, Y.-W. Shia, D.-Y. Guo, W.-Y. Lee, Shear-enhanced stretchable polymer semiconducting blends for polymer-based field-effect transistors. *Macromol. Res.* **28**, 660–669 (2020).

54. S. Georgakopoulos, F. G. del Pozo, M. Mas-Torrent, Flexible organic transistors based on a solution-sheared PVDF insulator. *J. Mater. Chem. C* **3**, 12199–12202 (2015).

55. S. H. Lee, D. G. Lee, H. Jung, S. Lee, Application of calendering for improving the electrical characteristics of a printed top-gate, bottom-contact organic thin film transistors. *Jpn. J. Appl. Phys.* **57**, 05GC01 (2018).

56. J.-M. Verilhac, M. Benwadih, A.-L. Seiler, S. Jacob, C. Bory, J. Bablet, M. Heitzman, J. Tallal, L. Barbut, P. Frère, G. Sicard, R. Gwoziecki, I. Chartier, R. Coppard, C. Serbutoviez, Step toward robust and reliable amorphous polymer field-effect transistors and logic functions made by the use of roll to roll compatible printing processes. *Org. Electron.* **11**, 456–462 (2010).

57. M. M. Voigt, A. Guite, D.-Y. Chung, R. U. A. Khan, A. J. Campbell, D. D. C. Bradley, F. Meng, J. H. G. Steinke, S. Tierney, I. McCulloch, H. Penxten, L. Lutsen, O. Douheret, J. Manca, U. Brokmann, K. Sönnichsen, D. Hülsenberg, W. Bock, C. Barron, N. Blanckaert, S. Springer, J. Grupp, A. Mosley, Polymer field-effect transistors fabricated by the sequential gravure printing

of polythiophene, two insulator layers, and a metal ink gate. *Adv. Funct. Mater.* **20**, 239–246 (2010).

58. J. Kim, T. Hassinen, W. H. Lee, S. Ko, Fully solution-processed organic thin-film transistors by consecutive roll-to-roll gravure printing. *Org. Electron.* **42**, 361–366 (2017).

59. S. Sung, W.-J. Lee, M. M. Payne, J. E. Anthony, C.-H. Kim, M.-H. Yoon, Large-area printed low-voltage organic thin film transistors via minimal-solution bar-coating. *J. Mater. Chem. C* **8**, 15112–15118 (2020).

60. D. W. Kim, S.-Y. Min, Y. Lee, U. Jeong, Transparent flexible nanoline field-effect transistor array with high integration in a large area. *ACS Nano* **14**, 907–918 (2020).

61. K. Liu, Y. Bian, J. Kuang, Q. Li, Y. Liu, W. Shi, Z. Zhao, X. Huang, Z. Zhu, Y. Guo, Y. Liu, Carbon nanotube-based van der Waals heterojunction electrodes for high-performance intrinsically stretchable organic photoelectric transistors. *Giant* **7**, 100060 (2021).

62. M. Y. Lee, Y. Oh, J. Hong, S. J. Lee, D. G. Seong, M.-K. Um, J. H. Oh, Fabrication of stretchable and transparent core–shell polymeric nanofibers using coaxial electrospinning and their application to phototransistors. *Adv. Electron. Mater.* **7**, 2001000 (2021).

63. T. U. Nam, J. H. Jeong, N. T. P. Vo, M. W. Jeong, J. H. Ma, M. H. Park, J. Park, S. J. Kang, J. Y. Oh, Intrinsically stretchable phototransistors with polymer-QD-polymer multi-layered hybrid films for visible-NIR perspective electronic skin sensors. *Chem. Eng. J.* **492**, 152143 (2024).

64. S. Guo, H. Xu, Y. Zheng, L. Li, Z. Li, L. Zhang, H. Zhang, X. Wang, J. Li, L. Wang, L. Liu, Z. Lou, Stretchable reconfigurable logic gate based on near-infrared photoelectric modulation. *Nano Energy* **110**, 108361 (2023).

65. H. Yu, X. Zhao, M. Tan, J. Li, M. Zhang, G. Zhao, Y. Fu, P. Xue, Y. Shi, Y. Tong, Q. Tang, Y. Liu, Dual-channel co-regulated organic phototransistors for ultrasensitive near-infrared biomimetic eyes. *Adv. Mater.* **38**, e09485 (2026).

66. M. Liu, H. Wang, Q. Tang, X. Zhao, Y. Tong, Y. Liu, Ultrathin air-stable n-type organic phototransistor array for conformal optoelectronics. *Sci. Rep.* **8**, 16612 (2018).

67. T.-Y. Kim, J. Ha, K. Cho, J. Pak, J. Seo, J. Park, J.-K. Kim, S. Chung, Y. Hong, T. Lee, Transparent large-area MoS_2 phototransistors with inkjet-printed components on flexible platforms. *ACS Nano* **11**, 10273–10280 (2017).

68. C. Lee, H. Kim, Y. Kim, Short-wave infrared organic phototransistors with strong infrared-absorbing polytriarylamine by electron-transfer doping. *Npj Flex. Electron.* **5**, 10 (2021).

69. M. Y. Lee, J. Hong, E. K. Lee, H. Yu, H. Kim, J. U. Lee, W. Lee, J. H. Oh, Highly flexible organic nanofiber phototransistors fabricated on a textile composite for wearable photosensors. *Adv. Funct. Mater.* **26**, 1445–1453 (2016).

70. H. Han, S. Nam, J. Seo, C. Lee, H. Kim, D. D. C. Bradley, C.-S. Ha, Y. Kim, Broadband all-polymer phototransistors with nanostructured bulk heterojunction layers of NIR-sensing n-type and visible light-sensing p-type polymers. *Sci. Rep.* **5**, 16457 (2015).

71. J. Zhong, X. Wu, S. Lan, Y. Fang, H. Chen, T. Guo, High performance flexible organic phototransistors with ultrashort channel length. *ACS Photonics* **5**, 3712–3722 (2018).

72. J. Huang, J. Du, Z. Cevher, Y. Ren, X. Wu, Y. Chu, Printable and flexible phototransistors based on blend of organic semiconductor and biopolymer. *Adv. Funct. Mater.* **27**, 1604163 (2017).

73. P. Hao, W. Jiang, M. Li, M. Li, Q. Liu, L. Yang, Y. Tan, Y. Wang, D. Wang, Fully flexible yarn-based phototransistors for UV–visible light detection. *ACS Photonics* **11**, 128–138 (2024).

74. H. Lee, J. Kim, H. Kim, J. Kim, S. Kwon, Colour-barcoded magnetic microparticles for multiplexed bioassays. *Nat. Mater.* **9**, 745–749 (2010).

75. J. Lee, P. W. Bisso, R. L. Srinivas, J. J. Kim, A. J. Swiston, P. S. Doyle, Universal process-inert encoding architecture for polymer microparticles. *Nat. Mater.* **13**, 524–529 (2014).

76. B. Yoo, C. Ryu, S. Lee, S. Jeong, Y. You, D. Baek, D. Kim, I. Jeon, K.-S. An, J. Oh, J. Lee, High-throughput multiplexed plasmonic color encryption of microgel architectures via programmable dithering-mask flow microlithography. *Adv. Mater.* **37**, e2405388 (2025).

77. M.-J. Sun, Y. Liu, Y. Yan, R. Li, Q. Shi, Y. S. Zhao, Y.-W. Zhong, J. Yao, In situ visualization of assembly and photonic signal processing in a triplet light-harvesting nanosystem. *J. Am. Chem. Soc.* **140**, 4269–4278 (2018).

78. Q. Lv, X.-D. Wang, Y. Yu, C.-F. Xu, Y.-J. Yu, X.-Y. Xia, M. Zheng, L.-S. Liao, Lateral epitaxial growth of two-dimensional organic heterostructures. *Nat. Chem.* **16**, 201–209 (2024).

79. S. R. Nicewarner-Peña, R. G. Freeman, B. D. Reiss, L. He, D. J. Peña, I. D. Walton, R. Cromer, C. D. Keating, M. J. Natan, Submicrometer metallic barcodes. *Science* **294**, 137–141 (2001).

80. C. D. Keating, M. J. Natan, Striped metal nanowires as building blocks and optical tags. *Adv. Mater.* **15**, 451–454 (2003).

81. D. Widerker, G. V. Kaigala, M. Bercovici, Additive manufacturing of multi-metal microstructures by localized electrochemical deposition under hydrodynamic confinement. *Adv. Mater. Technol.* **9**, 2301290 (2024).

82. Y. Liu, F. Han, F. Li, Y. Zhao, M. Chen, Z. Xu, X. Zheng, H. Hu, J. Yao, T. Guo, W. Lin, Y. Zheng, B. You, P. Liu, Y. Li, L. Qian, Inkjet-printed unclonable quantum dot fluorescent anti-counterfeiting labels with artificial intelligence authentication. *Nat. Commun.* **10**, 2409 (2019).

83. K. Zub, S. Hoeppener, U. S. Schubert, Inkjet printing and 3D printing strategies for biosensing, analytical, and diagnostic applications. *Adv. Mater.* **34**, e2105015 (2022).

84. K. Braeckmans, S. C. De Smedt, C. Roelant, M. Leblans, R. Pauwels, J. Demeester, Encoding microcarriers by spatial selective photobleaching. *Nat. Mater.* **2**, 169–173 (2003).

85. C. Huang, B. Lucas, C. Vervaet, K. Braeckmans, S. Van Calenbergh, I. Karalic, M. Vandewoestyne, D. Deforce, J. Demeester, S. C. De Smedt, Unbreakable codes in electrospun

fibers: Digitally encoded polymers to stop medicine counterfeiting. *Adv. Mater.* **22**, 2657–2662 (2010).

86. R. Catalano, Y. Zhao, M. Pecak, T. Korten, S. Diez, Barcoding microtubules: Encoding information onto macromolecules by photobleaching. *Nano Lett.* **25**, 5283–5290 (2025).
